# A single class of ARF GTPase activated by several pathway-specific ARF-GEFs regulates essential membrane traffic in *Arabidopsis*

**DOI:** 10.1371/journal.pgen.1007795

**Published:** 2018-11-15

**Authors:** Manoj K. Singh, Sandra Richter, Hauke Beckmann, Marika Kientz, York-Dieter Stierhof, Nadine Anders, Florian Fäßler, Michael Nielsen, Christian Knöll, Alexis Thomann, Mirita Franz-Wachtel, Boris Macek, Karen Skriver, Peter Pimpl, Gerd Jürgens

**Affiliations:** 1 Center for Plant Molecular Biology (ZMBP), Developmental Genetics, University of Tübingen, Tübingen, Germany; 2 Center for Plant Molecular Biology (ZMBP), Microscopy, University of Tübingen, Tübingen, Germany; 3 Section for Biomolecular Sciences, Department of Biology, Copenhagen Biocenter, University of Copenhagen, Copenhagen, Denmark; 4 Proteome Center Tübingen, University of Tübingen, Tübingen; University of Basel, SWITZERLAND

## Abstract

In eukaryotes, GTP-bound ARF GTPases promote intracellular membrane traffic by mediating the recruitment of coat proteins, which in turn sort cargo proteins into the forming membrane vesicles. Mammals employ several classes of ARF GTPases which are activated by different ARF guanine-nucleotide exchange factors (ARF-GEFs). In contrast, flowering plants only encode evolutionarily conserved ARF1 GTPases (class I) but not the other classes II and III known from mammals, as suggested by phylogenetic analysis of ARF family members across the five major clades of eukaryotes. Instead, flowering plants express plant-specific putative ARF GTPases such as ARFA and ARFB, in addition to evolutionarily conserved ARF-LIKE (ARL) proteins. Here we show that all eight ARF-GEFs of *Arabidopsis* interact with the same ARF1 GTPase, whereas only a subset of post-Golgi ARF-GEFs also interacts with ARFA, as assayed by immunoprecipitation. Both ARF1 and ARFA were detected at the Golgi stacks and the trans-Golgi network (TGN) by both live-imaging with the confocal microscope and nano-gold labeling followed by EM analysis. ARFB representing another plant-specific putative ARF GTPase was detected at both the plasma membrane and the TGN. The activation-impaired form (T31N) of ARF1, but neither ARFA nor ARFB, interfered with development, although ARFA-T31N interfered, like ARF1-T31N, with the GDP-GTP exchange. Mutant plants lacking both ARFA and ARFB transcripts were viable, suggesting that ARF1 is sufficient for all essential trafficking pathways under laboratory conditions. Detailed imaging of molecular markers revealed that ARF1 mediated all known trafficking pathways whereas ARFA was not essential to any major pathway. In contrast, the hydrolysis-impaired form (Q71L) of both ARF1 and ARFA, but not ARFB, had deleterious effects on development and various trafficking pathways. However, the deleterious effects of ARFA-Q71L were abolished by ARFA-T31N inhibiting cognate ARF-GEFs, both in *cis* (ARFA-T31N,Q71L) and in *trans* (ARFA-T31N + ARFA-Q71L), suggesting indirect effects of ARFA-Q71L on ARF1-mediated trafficking. The deleterious effects of ARFA-Q71L were also suppressed by strong over-expression of ARF1, which was consistent with a subset of BIG1-4 ARF-GEFs interacting with both ARF1 and ARFA. Indeed, the SEC7 domain of BIG5 activated both ARF1 and ARFA whereas the SEC7 domain of BIG3 only activated ARF1. Furthermore, ARFA-T31N impaired root growth if ARF1-specific BIG3 was knocked out and only ARF1- and ARFA-activating BIG4 was functional. Activated ARF1 recruits different coat proteins to different endomembrane compartments, depending on its activation by different ARF-GEFs. Unlike ARF GTPases, ARF-GEFs not only localize at distinct compartments but also regulate specific trafficking pathways, suggesting that ARF-GEFs might play specific roles in traffic regulation beyond the activation of ARF1 by GDP-GTP exchange.

## Author summary

Membrane traffic plays an important role in cellular homeostasis, cell-cell communication, nutrient uptake and organismal interaction, delivering membrane proteins as well as secreted proteins to their sites of action and degradation. This requires sorting of proteins into forming membrane vesicles, which then bud from a donor compartment and eventually fuse with an acceptor compartment, releasing their cargo. Formation of vesicles is initiated by ARF GTPase activation through an interacting ARF guanine-nucleotide exchange factor (ARF-GEF) on the donor membrane, resulting in the recruitment of specific coat proteins. In mammals, different classes of ARF GTPases are activated by diverse ARF-GEFs. In contrast, much less is known in plants, although there appears to be only a single family of large ARF-GEFs, and only one ARF class represented by ARF1 isoforms is conserved. Instead, there are plant-specific putative ARFs, which however, are functionally uncharacterized. Here we show that only ARF1 is required for normal development of Arabidopsis and can mediate all specific trafficking pathways whereas the other putative ARFs are not essential. Furthermore, ARF1 interacts with all ARF-GEFs acting in specific trafficking pathways. Our results thus suggest that ARF-GEFs rather than ARFs likely contribute to the specificity of membrane trafficking in plants.

## Introduction

ARF GTPases and their guanine-nucleotide exchange factors (ARF-GEFs) play essential roles in the formation of membrane vesicles that transport cargo proteins from donor to acceptor compartments within the endomembrane system. Sometimes called molecular switches, ARF proteins cycle between an inactive GDP-bound form and an active GTP-bound form [1, 2]. In mammals, ARF GTPases are grouped in three classes. Class-I ARFs (Arf1-3) are activated by large ARF-GEF GBF1 and required for the recruitment of COPI coat protein complex to the Golgi stack, resulting in the formation of COPI-coated vesicles for retrograde traffic from the Golgi stack to the endoplasmic reticulum (ER; [3]). However, GBF1 can also activate the class-II GTPase Arf5 [4]. Mammalian BIG1 and BIG2 appear to activate Arf1 and Arf3 at the trans-Golgi network (TGN), recruiting adaptor protein complex AP-1 for the formation of clathrin-coated vesicles in TGN-plasma membrane secretory traffic [3]. Thus, class-I ARFs mainly regulate secretory traffic. Although class-I and class-II ARFs act interchangeably at the Golgi stack, Arf3 seems to play a specific role there [5]. In addition to the large ARF-GEFs, there are small ARF-GEFs such as cytohesin or ARNO, which activate Arf1 as well as the endocytosis-related GTPase Arf6, the only member of class III. However, Arf6 is also activated by a medium-sized Arf6-specific ARF-GEF named EFA6 [3, 4]. Besides ARFs, there are ARF-like (Arl) or Arf-related (Arfrp1) proteins, which perform diverse biological functions including formation of tubulin heterodimers [4, 6].

The situation is less clear in flowering plants. Early bioinformatic surveys of the *Arabidopsis* genome revealed 19 genes coding for ARF or ARF-LIKE (ARL) proteins [7–9]. Notably, class-I ARFs are conserved across the eukaryotes whereas class-II and class-III ARFs appear absent from plants. On the other hand, there are at least two additional seemingly plant-specific putative ARF classes named A and B, however their biological roles are unknown. Another peculiarity of plants is the absence of small and medium-sized ARF-GEFs, which play essential roles in ARF activation in non-plant eukaryotes [3, 10]. Instead, the conserved group of large ARF-GEFs has increased to three GBF1-like proteins (GN, GNL1 and GNL2) and 5 BIG1-like proteins (BIG1 to BIG5) in *Arabidopsis* [1, 2]). *Arabidopsis* ARF-GEFs localize at distinct compartments and regulate specific trafficking pathways. GN mediates polar recycling of the auxin-efflux carrier PIN1 from endosomes to the basal plasma membrane, resulting in polar auxin transport [11]. Both GN and GNL1 mediate retrograde traffic of COPI vesicles from the Golgi stack to the ER [12]. GNL2 is a pollen-specific counterpart of GN, which also can substitute for both GN and GNL1 when expressed from the *GN* promoter [13]. Of the 5 BIGs, BIG1-4 are functionally overlapping ARF-GEFs that mediate the late-secretory pathway from the TGN to the plasma membrane as well as trafficking to the vacuole and to the cell division plane [14]. BIG5 (also known as MIN7 and BEN1) is involved in pathogen response and in endosomal trafficking [15–18].

Here we address the problem of specificity in the interaction of ARFs with ARF-GEFs as regards the regulation of distinct trafficking pathways. Our results indicate that all ARF-GEFs of *Arabidopsis* interact with class-I ARFs and that only class-I ARFs, but not ARFs of the two other classes A and B, are essential for viability. Class-A ARFs are conserved within the plant lineage and appear to be activated by a subset of BIG ARF-GEFs with dual substrate specificity for ARFA and ARF1 but they are not vital. The single class-B ARF might have originated from an ARF1 gene duplication event late in flowering plant evolution, and blocking its activation has no deleterious effect. In conclusion, ARF-GEFs seem to play specific roles in traffic regulation beyond the activation of ARF1 by GDP-GTP exchange.

## Results

### ARF GTPases versus ARF-like GTPases encoded in the *Arabidopsis* genome

**Fig 1:**
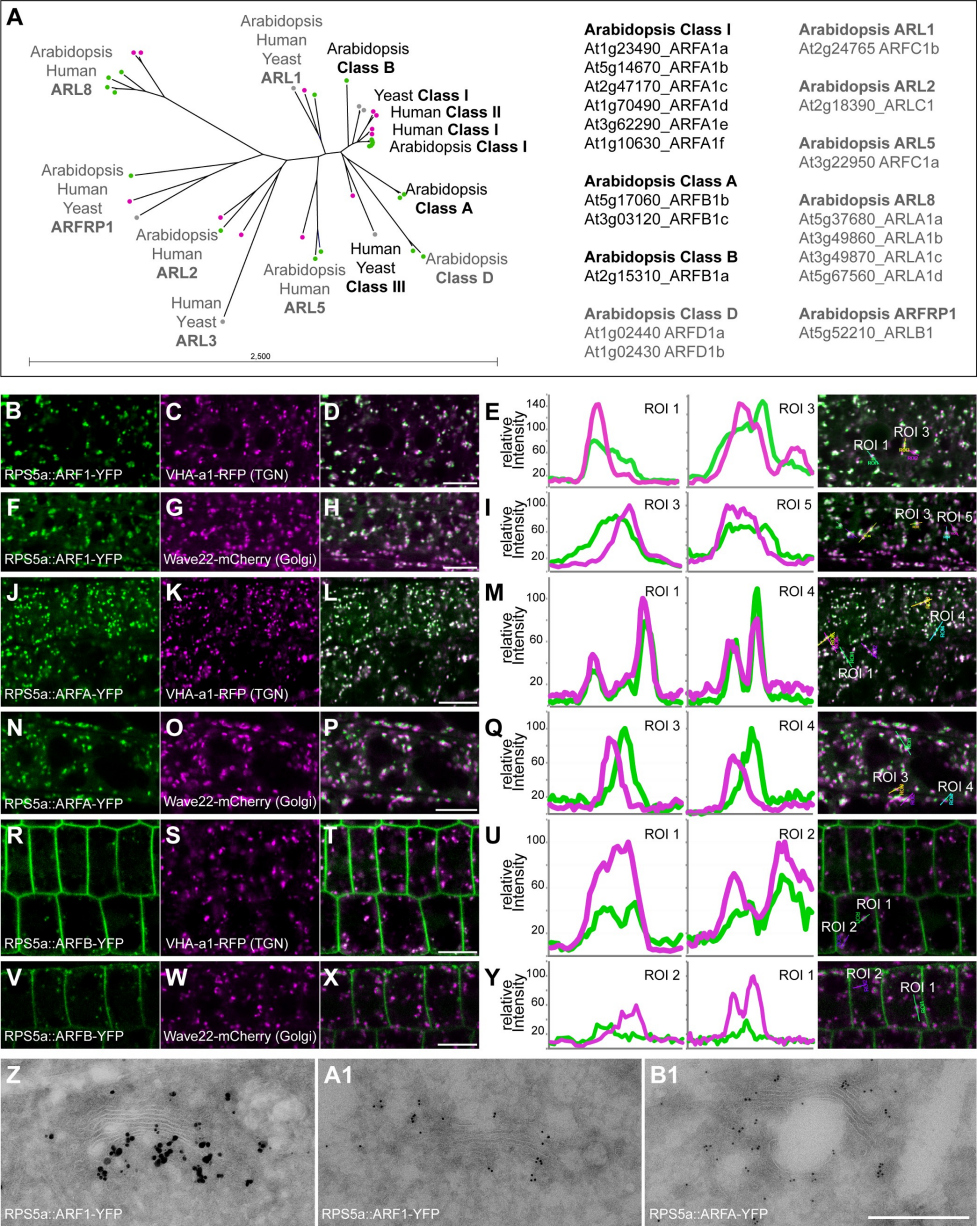
Phylogenetic tree of ARF GTPases and ARF-like proteins, and the subcellular localization of *Arabidopsis* ARF GTPases. (**A**) *Left*: Phylogenetic tree of ARFs and ARF-likes from *Arabidopsis* (At; green), Human (Hs; magenta) and Yeast (Sc; grey). Class I ARFs, ARL1/2/5/8 and ARFRP1 are conserved among eukaryotes, whereas ARF classes A, B and D are plant-specific. Class II and class III ARFs are present in human and yeast but not in plants. *Right*: List of Arabidopsis ARF and ARL GTPases with their gene identifiers. (**B-Y**) Subcellular localization by CLSM of YFP-tagged ARF-GTPases of classes I, A and B expressed from the *RPS5a* promoter (green channel). (**B-I**) Class-I ARF (ARFA1c; B; F) co-localized partially with TGN-marker VHA-a1-RFP (magenta; C; D) and Golgi-marker Wave22-mCherry (magenta; G; H). Co-localization in two regions of interest (ROI) in line intensity profiles (E, I). (**J-Q**) Class-A ARF (ARFB1b; J; N) co-localized partially with TGN-marker VHA-a1-RFP (magenta; K; L) and the Golgi-marker Wave22-mCherry (magenta; O; P). Co-localization in two regions of interest (ROI) in line intensity profiles (M, Q). (**R-Y**) Class-B ARF (R, V) localized at the plasma membrane and in intracellular punctae. ARFB co-localized partially with TGN-marker VHA-a1-RFP (magenta; S; T) but not with Golgi-marker Wave22-mCherry (magenta; W; X). Co-localization in two regions of interest (ROI) in line intensity profiles (U, Y). Scale bar, 10 μm. (**Z-B1**) Ultrastructural localization of YFP-tagged ARF GTPases ARF1 (Z, A1) and ARFA (B1) with gold-labelled anti-GFP antibodies. Silver-enhanced 1 nm gold (Z), 6 nm gold (A1-B1). Scale bar (Z-B1), 500 nm.

The *Arabidopsis* genome codes for 19 proteins annotated as ARF or ARF-LIKE (ARL) GTPases (Fig 1A and S1 Table; see also [8, 9]). To define ARFs as opposed to ARLs, we initially identified orthologs across all five major clades of eukaryotes (Archaeplastida, SAR, Excavata, Amoebozoa, Opisthokonta) because the counterparts in mammals and yeast have been functionally characterized (S1A Fig and S2 Table). This distinguished class-I ARFs (6 ARF1 isoforms) from 5 ARL classes comprising ARL1, ARL2, ARL5, ARL8 (4 isoforms), and ARFRP1 (Figs 1A and S1A and S1 and S2 Tables). For example, analysis of ARL2 function in *Arabidopsis* confirmed a conserved role of this protein in tubulin dimer formation [19]. Two ARLs were originally classified as ARFs: ARL1 was known as ARF3 (or ARFC1b) whereas ARL5 was named ARFC1(a) [8]. In contrast, the remaining 5 ARF-related proteins are largely confined to the plant lineage, functionally undefined and were considered candidate ARFs representing 3 different classes: 2 class-A ARFs, 1 class-B ARF, and 2 class-D ARFs (S1A Fig and S1 and S2 Tables). These classes differ in evolutionary age: Unlike class-I ARFs, class-A ARFs are restricted to the green lineage (Archaeplastida, although not present in Rhodophyceae), ranging from algae to flowering plants, but not present in the related eukaryotic clade of SAR, the other subgroup of Diaphoretickes (S1A Fig and S2 Table). Class-B ARFs originated late in dicot flowering plant evolution, being confined to the order of Brassicales which includes *Arabidopsis* (S1 Table). No ARFB-related sequences were identified in the basal angiosperm *Amborella*, the basal dicot *Aquilegia* or the secondarily simplified monocot *Spirodela* (S1 Table). There was an ARF sequence in the excavate *Giardia lamblia* (GL50803_7789) that grouped with Arabidopsis ARFB but was more closely related to ARF1 than ARFB (e-97 and e-78, respectively; S1A Fig and S2 Table). The two closely related class-D ARF proteins appear confined to the *Arabidopsis* family named Brassicaceae (S1 and S2 Tables), suggesting that ARFD originated only recently. Although we detected an *Ectocarpus* EsArlX with low sequence identity to both ARF1 and ARFD of *Arabidopsis*, there was no such ArlX in the other SAR subclades–diatoms, oomycetes and Alveolata (S1 Fig and S2 Table). With ARL1 being the eukaryotically conserved ARL protein most closely related to ARF1 by sequence, ARFs might be expected to be comparably related to ARF1 (blastP e-values: ARF1s among themselves, -102; the other groups to ARF1: ARFB, -70; ARL1, -65; ARFA, -63; ARL5, -55; ARFD, -46; ARL2, -39). Class-D proteins were excluded from further consideration because of their late origin in flowering plant evolution and their distant relatedness to ARF1. We thus focused our study on ARFA and ARFB, in addition to ARF1.

The 6 class-I ARFs share at least 97% sequence identity, suggesting redundant functions [20]. They are less divergent than the human class-I ARFs. Two Arabidopsis ARF1 isoforms–ARFA1a and ARFA1d –are identical in sequence. Moreover, the most divergent ARF1 isoform ARFA1b is expressed in haploid pollen and not in somatic tissues [21]. The remaining 4 isoforms of Arabidopsis ARF1s are polymorphic at amino acid residues 6 (2G, 2A) and 179 (2S, 1N, 1G). Consistently, all ARF1 isoforms were detected by immunostaining with specific antiserum raised against class-I member ARFA1c (designated ARF1A1c by TAIR; https://www.arabidopsis.org/), which also cross-reacted with ARF1 from maize [22]. ARF1-YFP was localized by live-cell imaging and double labeling with specific markers to the Golgi stacks and trans-Golgi networks (TGN) (Fig 1B–1I; see S1C Fig for quantitative assessment of co-localization), which was also confirmed by immunogold labeling and EM localization (Fig 1Z and 1A1) and by immunolocalization of ARF1 with anti-ARF1 antiserum [23]. The two class-A members also share high sequence identity with each other (94%), suggesting redundancy within this class. ARFA-YFP was localized to the TGN and, less strongly, Golgi stacks by live-cell imaging and by immunogold labeling and EM localization (Figs 1J–1Q and 1B1 and S1C). The single class-B ARF fused to YFP (ARFB-YFP) was detected at the plasma membrane and also co-localized with the TGN marker VHA-a1-RFP but not with the Golgi marker wave22-mCherry (Figs 1R–1Y and S1C; see also an earlier report on plasma-membrane localization of ARFB-YFP transiently expressed in tobacco leaf cells and protoplasts [24]). Thus, ARF1 and ARFA overlapped in their detectable subcellular localization at the Golgi stacks and the TGN whereas ARFB appeared to be present at both the plasma membrane and the TGN.

### Interaction of *Arabidopsis* ARF GTPases with ARF-GEFs

Unlike ARLs, true ARFs are activated by ARF-GEFs. We therefore assayed ARF1, ARFA and ARFB for their ability to interact with the previously characterized *Arabidopsis* ARF-GEFs (S1B Fig; [1, 11–14]). To explore the presumed interactions in planta, we performed immunoprecipitation experiments in the presence of the fungal toxin brefeldin A (BFA) with YFP-tagged ARFs representing the three classes I, A and B plus YFP-tagged RABG3f GTPase as a control for non-specific interaction, and subjected the immunoprecipitates to mass spectrometry (S3 Table). BFA is known to inhibit the GDP-GTP exchange reaction, freezing the abortive complex of ARF•GDP with BFA-sensitive ARF-GEF on the membrane [25]. Analysis of the data revealed that ARF1 interacted with all ARF-GEFs expressed in seedlings (BIG1-5, GN and GNL1 but excluding pollen-specific GNL2) whereas ARFA only interacted with BIG5. ARFB appeared to interact with BIG5 and, less strongly, also with BIG1 and BIG2. Only background levels of ARF-GEFs were detected in the RABG3f immunoprecipitate which instead contained RAB-GDI and retromer subunit VPS35 isoforms A and B (S3 Table). Thus, the interaction data suggest that conserved ARF1 is likely activated by all ARF-GEFs whereas ARFA and ARFB might be activated by a specific subset of post-Golgi ARF-GEFs.

### Are ARF proteins of the different classes essential?

**Fig 2:**
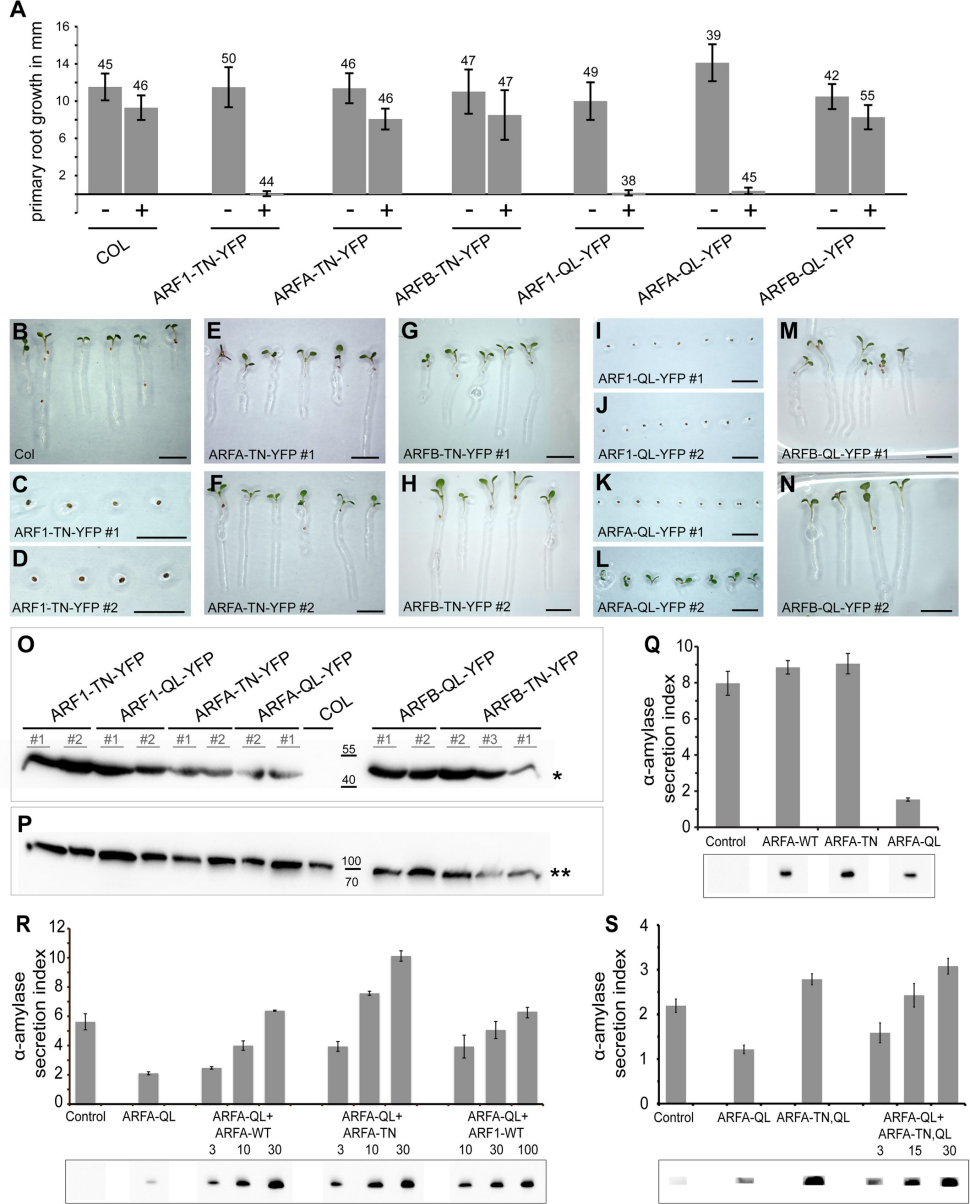
ARF1 but not ARFA or ARFB is crucial for primary root growth and seed germination. (**A-P**) Activation-impaired (T31N; TN) or hydrolysis-impaired (Q71L; QL) variants of ARF1, ARFA and ARFB were expressed from the estradiol-inducible system. (**A**) Primary root growth was severely affected by ARF1-TN-YFP, ARF1-QL-YFP and ARFA-QL-YFP but not by ARFA-TN-YFP, ARFB-TN-YFP or ARFB-QL-YFP. Number of seedlings analyzed is indicated above each column. (+), 20μM estradiol; (-), without estradiol. See also S1 Data. (**B-N**) Seed germination on estradiol-containing medium is inhibited by ARF1-TN-YFP (C, D), ARF1-QL-YFP (I, J) and ARFA-QL-YFP (K, L) but not by ARFA-TN-YFP (E, F), ARFB-TN-YFP (G, H) or ARFB-QL-YFP (M, N). Scale bar, 5 mm (B-N). (**O, P**) Western blot of estradiol-induced expression of activation-impaired (TN) or hydrolysis-impaired (QL) variants of ARF1, ARFA and ARFB in seedlings (O). Asterisk, expected size of approx. 47kDa. (P) γCOP as loading control; two asterisks, expected size of approx. 100kDa. COL, wild-type control. (**Q-S**) Quantitative secretion assay. (**Q**) Co-expression of the secretory reporter α-amylase with wild type (WT), activation-impaired (TN) or hydrolysis-impaired (QL) ARFA variants in tobacco protoplasts. Secretion index, ratio of extracellular to intracellular reporter activity; control, expression of α-amylase alone. **(R)** Co-expression of α-amylase and ARFA-QL with rising concentrations of ARFA-WT, ARFA-TN or ARF1-WT. Numbers below indicate the amount of respective plasmid used for protoplast transformation relative to ARFA-QL; constant amounts of α-amylase and ARFA-QL were transformed. **(S)** Co-expression of α-amylase and ARFA-QL with rising concentrations of ARFA-TN,QL. Numbers below indicate the amount of respective plasmid used for protoplast transformation relative to ARFA-QL; constant amounts of α-amylase and ARFA-QL were transformed. Panels below (**Q-S**): ARF protein expression levels evidenced indirectly by detection of GFP. Both *ARF* and *GFP* coding sequences are under control of the bidirectional *mas* promoter (consisting of a mas1’ and a mas2’ part) on the same plasmid, with mas1’ directing GFP expression and mas2’ directing ARF expression in a ratio of 1 to 10 [65]. Note full recovery of secretion by ARFA-TN (**R**) and ARFA-TN,QL (**S**) overexpression. See also S2, S3 and S4 Data.

To determine the function of the various ARF classes, we generated transgenic plants overexpressing ARF-T31N-YFP or ARF-Q71L-YFP in an inducible or conditional manner. The T31N mutant ARF GTPases are unable to undergo GDP-GTP exchange. These inactive ARFs will bind tightly to, and thus block, cognate activating ARF-GEFs. Since ARF-GEFs are limiting, overexpression of ARF-T31N will titrate out interacting ARF-GEFs. In contrast, ARF-Q71L is thought to slow down GTP hydrolysis, keeping the ARF GTPase in an active, GTP-bound form. Active ARF GTPases will interact with effector proteins and likely titrate out interacting coat proteins by blocking the uncoating process [26]. Both ARF variants should thus have deleterious, albeit different, effects on membrane trafficking. Using an estradiol (Est)-inducible promoter [27], we analyzed the effects of ARF1, ARFA or ARFB on seedling root growth and seed germination (Fig 2A–2P). When transferred from -Est to +Est plates the roots of ARF1-T31N-YFP seedlings did not grow anymore whereas root growth of ARFA-T31N-YFP and ARFB-T31N-YFP seedlings was not affected, although the latter two variants were also well expressed (Fig 2A, 2O–2P). The analysis of seed germination resulted in similar effects (Fig 2B–2N). ARF1-T31N-YFP seeds did not germinate on +Est plates whereas germination of ARFA-T31N-YFP and ARFB-T31N-YFP seeds was unaffected (Fig 2B–2D, compare with 2E, 2F, 2G–2H). In conclusion, only ARF1-T31N-YFP overexpression caused clear-cut mutant phenotypes, suggesting that only ARF1 is essential for normal development.

Hydrolysis-impaired ARF1-Q71L-YFP has been used to interfere with secretory and vacuolar traffic in plant cells [28–30]. Est-inducible ARFB-Q71L-YFP was well expressed but had no adverse effects on seedling root growth nor on seed germination (Fig 2A, 2M, 2N, 2O and 2P). In contrast, *Est>>ARFA-Q71L-YFP* seed germination was (largely) blocked on estradiol plates, which resembled the situation in *Est>>ARF1-T31N-YFP* (Fig 2K and 2L, compare with Fig 2C and 2D) or when secretion was inhibited as a result of inactivation of early or late secretory ARF-GEFs, respectively [12, 14]. In addition, root growth of *Est>>ARFA-Q71L-YFP* transgenic seedlings was blocked upon transfer to estradiol plates (Fig 2A). Thus, ARFA-Q71L-YFP had developmental consequences comparable to ARF1-T31N-YFP and inactivation of ARF-GEFs GNL1 and GN or BIG1-4. Interestingly, ARF1-Q71L-YFP caused essentially the same developmental defects as did ARF1-T31N-YFP or ARFA-Q71L-YFP (Fig 2A, 2I and 2J, compare with Fig 2C, 2D, 2K and 2L).

Since ARFA and ARF1 had strong influence on seedling development, we tested whether expression of inhibitory mutant forms of ARFA and ARF1 variants also affects embryo development. Therefore, we expressed the T31N and Q71L forms with the *GAL4>>UAS* two-component expression system involving the strong *RPS5A* ribosomal protein gene promoter [31]. ARF1-T31N-YFP caused early embryo arrest, with cells displaying cytokinesis defects, which indicates an essential role of ARF1-mediated trafficking from fertilization onward (S2A–S2L Fig). In contrast, *RPS5A*-driven overexpression of ARFA-T31N-YFP had no obvious phenotypic effects in embryogenesis (S2M–S2O Fig). Two-component expression of either ARF1-Q71L-YFP or ARFA-Q71L-YFP interfered with embryogenesis, although with some delay as compared to the early effect of ARF1-T31N-YFP (S2P–S2U and S2V–S2X Fig, compare with S2G–S2L Fig). These data indicate that ARF1 plays a crucial role in embryo development. In contrast, ARFA might not be essential since expression of ARFA-T31N-YFP had no influence on embryo development.

To address the lack of deleterious effects in ARFA-T31N and ARFB-T31N, we analyzed knockout or nearly complete knockdown mutants. Single and double knockouts of the two *ARFA* genes by T-DNA insertion did not cause any major abnormality (S2Y and S2Z Fig). No such knockout was available for the single *ARFB* gene. Instead, six transgenic lines expressing artificial microRNA (amiRNA) directed against *ARFB* mRNA from the *RPS5A* promoter were generated (*RPS5A*::*amiR(ARFB)*) and all those lines showed a strong reduction of *ARFB* transcript level whereas *ARFA* mRNA was not affected (S2B1 Fig). However, the strong reduction of ARFB function did not result in any morphological abnormality (S2A1 Fig). These observations suggest that both class-A and class-B ARFs have no essential roles. To explore the possibility that ARFA and ARFB might have overlapping functions, we strongly co-expressed ARFA-T31N-YFP and ARFB-T31N-YFP in the same plants, which, however, did not yield any obvious mutant phenotype (S2C1 Fig). The same lack of deleterious phenotypic effect was also observed in mutant plants lacking *ARFA* transcripts because of genetic double knockout and *ARFB* transcripts because of expression of artificial microRNA (*RPS5A*::*amiR(ARFB)*) (S3 Fig). In conclusion, only ARF1 appears to be directly required for essential endomembrane traffic in plants whereas abolishing the activity of both ARFA and ARFB had no detectable effect.

Since ARFA-T31N had no obvious biological effect we tested whether the T31N mutation behaved as expected by inhibiting cognate ARF-GEF(s) that mediate ARFA activation. To this end, we transiently expressed mutant versions–T31N and Q71L –of class-A ARF GTPase in protoplasts and analyzed them for inhibitory effects in a quantitative secretion assay, as had been done for ARF1 [30]. In our secretion assay, ARFA-Q71L inhibited secretory trafficking whereas ARFA-T31N had no inhibitory effect (Fig 2Q). To clarify why ARFA-T31N had no effect in contrast to ARFA-Q71L, we co-expressed rising concentrations of activation-impaired ARFA-T31N together with a constant amount of hydrolysis-impaired ARFA-Q71L. This resulted in concentration-dependent suppression of the inhibitory effect of ARFA-Q71L, with 10-fold excess of ARFA-T31N fully restoring secretion to the control level (Fig 2R). These observations suggest that ARFA-T31N is likely to block the activation of ARFA-Q71L by cognate ARF-GEFs. To substantiate this conclusion, we tested an ARFA-T31N,Q71L double mutant construct in the quantitative secretion assay. This double mutant did not interfere with secretion of amylase (Fig 2S). Moreover, the same double mutant also suppressed the inhibitory effect of ARFA-Q71L in a concentration-dependent manner (Fig 2S). We then analyzed the in-planta effects of *Est>>ARFA-T31N*,*Q71L-YFP* double mutant transgene. In contrast to the *Est>>ARFA-Q71L-YFP* single mutant, the double mutant did not interfere with any developmental processes when induced by estradiol treatment (S4 Fig). Thus, ARFA might act in a non-essential trafficking pathway. Alternatively, ARFA activation might be carried out by ARF-GEFs with dual substrate specificity for ARFA and ARF1 and these overlap functionally with ARF-GEFs that do not activate ARFA but only ARF1 and thus cannot be inhibited by overexpression of ARFA-T31N. This latter idea is supported by the observation that rising concentrations of ARF1 suppressed the inhibitory effect of ARFA-Q71L on secretion in the protoplast assay (Fig 2R). Additional support comes from the analysis of the interaction of ARFs with ARF-GEFs and in-vitro GDP-GTP exchange assays (see below).

### Trafficking pathways affected by estradiol-induced overexpression of ARF1-T31N but not ARFA-T31N

**Fig 3:**
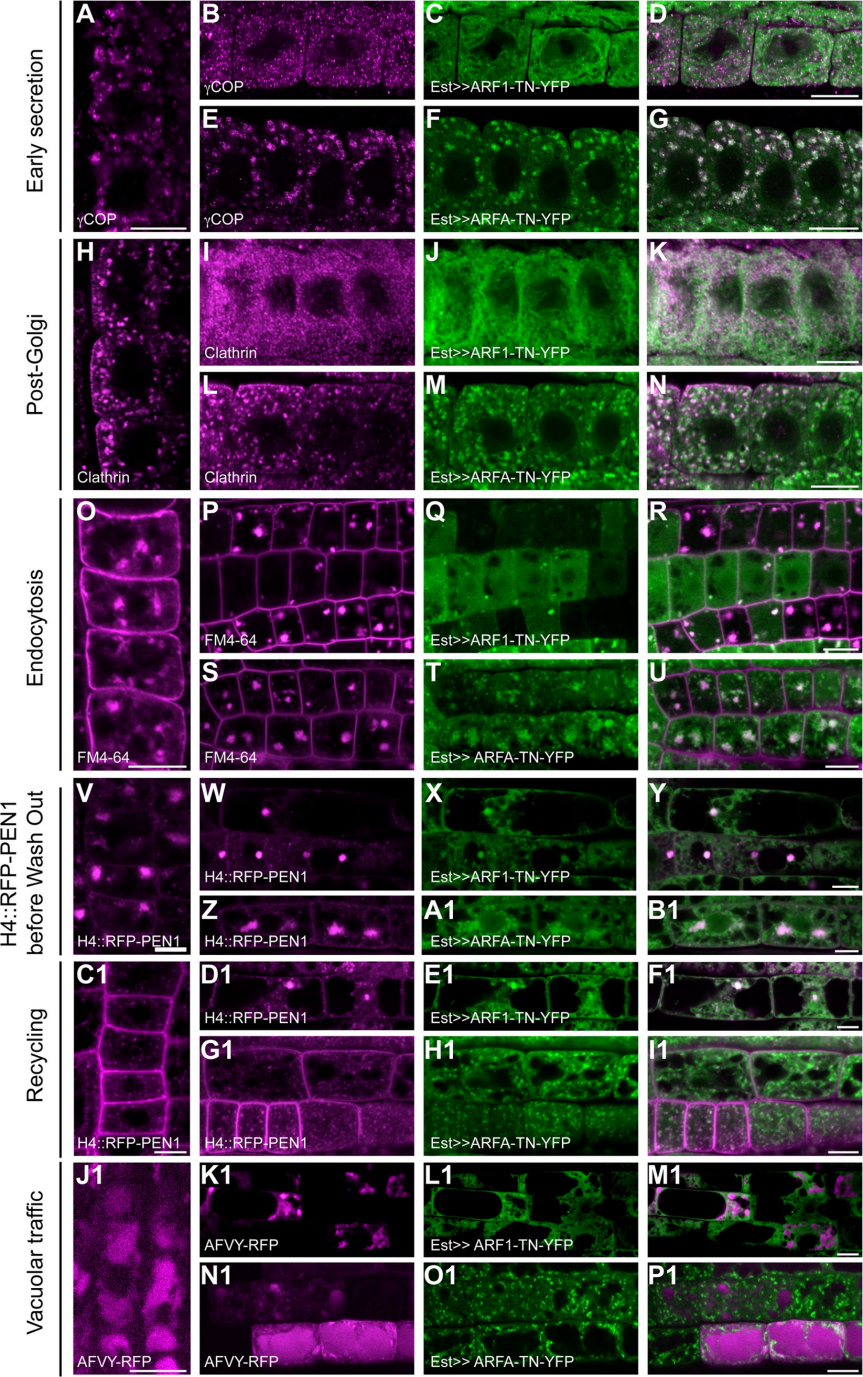
ARF1 but not ARFA regulates early secretion and post-Golgi trafficking. Expression of activation-impaired, YFP-tagged T31N (TN; green) variants of ARF1 and ARFA was induced by 20μM estradiol for 5-6h, and trafficking markers (magenta) were analyzed in immunostaining. (**A-G**) The COPI subunit γCOP (A, B, E) was recruited to the Golgi in wild-type control (A; Col) and in ARFA-TN-YFP (E-G) but stayed in the cytosol in ARF1-TN-YFP (B-D) expressing lines. (**H-N**) Clathrin coat (H, J, M) was recruited to the TGN in wild-type control (H) and in ARFA-TN-YFP (L-N) but stayed in the cytosol in ARF1-TN-YFP (I-K). (**O-U**) BFA treatment (50μM for 1h) was used to visualize endocytosed FM4-64 (O, P, S) in BFA compartments. Endocytosis was unaffected in wild-type control (O) and in ARFA-TN-YFP (S-U). In contrast, ARF1-TN-YFP (Q) interfered with endocytosis of FM4-64 (P-R). Note, cells expressing ARF1 strongly showed almost no endocytosed FM4-64, whereas cells with no or very low expression of ARF1-TN-YFP showed endocytosis. (**V-I1**) Recycling to the plasma membrane of RFP-PEN1 expressed from the *Histone 4* (*H4*) promoter after accumulation in BFA compartments. (**V-B1**) Seedlings were treated with 20μM estradiol and 50μM BFA for 5h. RFP-PEN1 (V, W, Z) localized in BFA compartments of root cells in wild-type (V), ARF1-TN-YFP (W-Y) and ARFA-TN-YFP (Z-B1). (**C1-I1**) Recycling of RFP-PEN1 was analyzed after BFA wash-out for 2h. RFP-PEN1 (C1, D1, G1) returned to the plasma membrane in wild-type controls (C1) and in ARFA-TN-YFP (G1-I1) but not in ARF1-TN-YFP (D1-F1). (**J1-P1**) Trafficking of the vacuolar cargo, AFVY-RFP (J1, K1, N1), expressed from the estradiol-inducible system, was inhibited in ARF1-TN-YFP (K1-M1) lines but not in wild-type (J1) or in ARFA-TN-YFP (N1-P1). Scale bar, 10μm.

**Fig 4:**
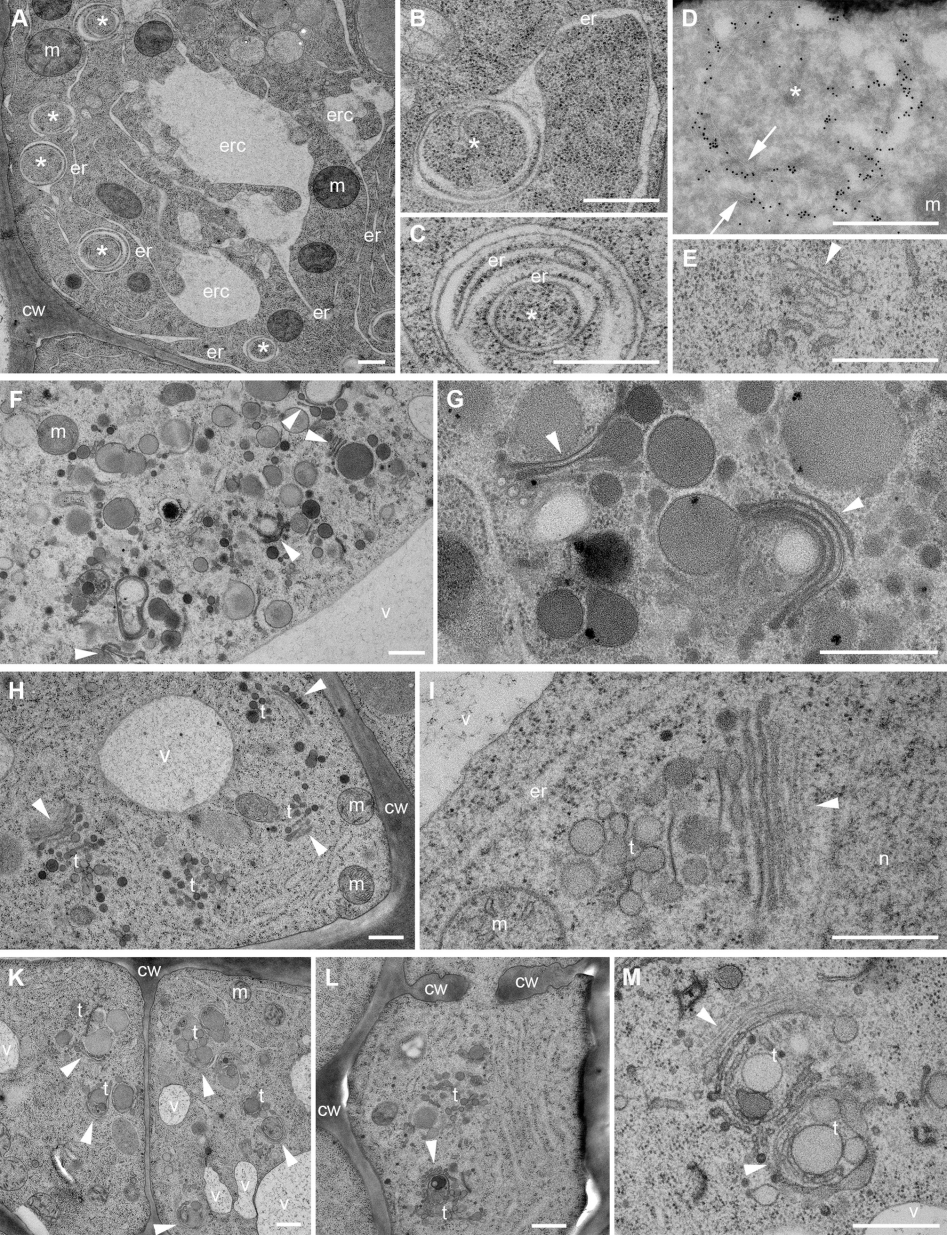
Ultrastructural defects of endomembrane compartments caused by interference with ARF1 or ARFA function. Seedling root cells were ultrastructurally analyzed after high-pressure freezing, freeze-substitution and embedding in epoxy resin. (**A-E**) ARF1-TN-YFP. (**A**) Tubular ER (er) connected to large compartments (erc) and circular structures (*). (**B, C**) Circular structures (*) connected to ER contain several membranes including ER membranes. (**D**) Circular structures are heavily labeled with gold-conjugated anti-ARF1 antibodies (arrows). (**E**) Disintegrated Golgi stack (arrowhead). (**F, G**) ARF1-QL-YFP. (**F**) Large vesicle aggregates with Golgi(-derived) cisternae (arrowheads). (**G**) Golgi cisternae (arrowheads) producing unusually large (secretory) vesicles. (**H, I**) ARFA-TN-YFP. (**H**) Golgi stacks (arrowheads) and TGN structures (t). (**I**) Golgi stack (arrowhead) with slightly enlarged TGN compartment (t) at higher magnification. (**K-M**) ARFA-QL-YFP. (**K**) Aberrant Golgi stacks (arrowheads) and TGN structures (t) in two adjacent cells. (**L**) Cell-wall stubs (cw) and aberrant Golgi stack (arrowhead) and TGN structures (t). (**M**) Aberrant rounded Golgi stacks (arrowheads) with budding vesicles and large TGN structures (t) at higher magnification. Abbreviations: cw, cell wall; er, endoplasmic reticulum (ER); erc, ER-connected compartment; m, mitochondrion; n, nucleus; t, trans-Golgi network (TGN); v, vacuole. Scale bars, 500 nm.

We analyzed the effects of the activation-impaired forms ARF1-T31N-YFP and ARFA-T31N-YFP in trafficking pathways that require specific ARF-GEFs. Est-induced expression of ARF1-T31N interfered with early secretory traffic. The COPI subunit γCOP remained cytosolic and the Golgi marker N-ST-YFP was relocated to the ER (Figs 3A–3D and S5A and S5B). These defects resembled the outcome of treating ARF-GEF knockout mutant *gnl1* with BFA to inactivate the functionally overlapping BFA-sensitive ARF-GEF GNOM in Golgi-ER retrograde trafficking [12]. Blocking of retrograde Golgi-ER traffic results in interference with anterograde secretory traffic. Consistently, plasma membrane-targeted SNARE protein SYP132 was trapped in the ER in ARF1-T31N-expressing lines (S5C–S5F Fig). Similarly, the cytokinesis-specific Qa-SNARE KNOLLE was trapped in the ER, rather than accumulating at the forming cell plate, which resulted in binucleate cells (S5J–S5M Fig). In addition, secretory-mCherry-HDEL, which was held back in the ER lumen, revealed abnormal morphology of the ER, comparable to the appearance of ER in the *gnl1* mutant allele *ermo1* (S5R–S5U Fig; [32, 33]). Ultrastructural analysis of cells overexpressing ARF1-T31N confirmed abnormalities of ER organization and disintegration of Golgi stacks, which resembled the ultrastructural phenotype of BFA-treated *gnl1* mutant cells (Fig 4A–4E; [12]). In contrast, ARFA-T31N-YFP overexpression had no deleterious effects on ER morphology or ER-Golgi traffic (Figs 3E–3G, 4H and 4I and S5G–S5I, S5N–S5Q, S5V–S5X).

ARF1-T31N-YFP also interfered with post-Golgi trafficking. The coat protein clathrin remained in the cytosol rather than associating with the TGN, which is consistent with the requirement of ARF-GEFs BIG1-4 for recruiting the clathrin adaptor-protein complex AP-1 to the TGN (Fig 3H–3K; [14]). Also, secretion of the polysaccharide xyloglucan, which is made in the Golgi stack and delivered to the extracellular space, was impaired, resulting in intracellular accumulation of xyloglucan (S6A–S6L Fig). Moreover, the TGNs were slightly enlarged, and there were additional TGNs (Fig 4I).

Endocytosis was also impaired by Est-induced overexpression of ARF1-T31N-YFP. BFA treatment was used to visualize the endocytosed lipophilic dye FM4-64, which accumulated in BFA compartments in wild-type seedling root cells but not in cells overexpressing ARF1-T31N-YFP (Fig 3O–3R). *H4*::*RFP-PEN1*, another marker for endocytosis, was retained at the plasma membrane in BFA-treated ARF1-T31N-YFP-expressing seedling roots, rather than accumulating in BFA compartments (S5Y–S5B1 Fig). In contrast, expression of ARFA-T31N-YFP had no deleterious effect on post-Golgi secretory or endocytic trafficking (Figs 3L–3N, 3S–3U and S6S–S6U).

The role of ARF1 in recycling was analyzed by treating seedlings with estradiol and BFA for 6 h followed by BFA washout for 2 h. In this way, endocytosed trafficking-marker protein first accumulated in the BFA compartments and subsequent washout of BFA would reveal either recycling to the plasma membrane or, if that was interfered with, retention of the marker in the BFA compartment. H4::RFP-PEN1 accumulated in BFA compartments before BFA washout (Fig 3V–3Y). After BFA washout, the marker still labeled endosomes in ARF1-T31N-expressing lines rather than being recycled to the plasma membrane (Fig 3C1–3F1).

A role of ARF1 in vacuolar trafficking was revealed by expression of the AFVY-RFP marker, which accumulates inside the vacuole in wild-type seedling root cells (Fig 3J1). Expression of ARF1-T31N-YFP blocked trafficking of AFVY-RFP to the vacuole (Fig 3K1–3M1). In contrast, ARFA-T31N-YFP blocked neither H4::RFP-PEN1 recycling nor vacuolar trafficking (Fig 3Z–3B1, 3G1–3I1 and 3N1–3P1).

In conclusion, overexpression of ARF1-T31N-YFP interfered with all known trafficking pathways including secretion, endocytosis, recycling or vacuolar traffic. In contrast, ARFA-T31N-YFP did not affect any of those pathways.

### Interference with trafficking by overexpression of ARF1-Q71L and ARFA-Q71L

**Fig 5:**
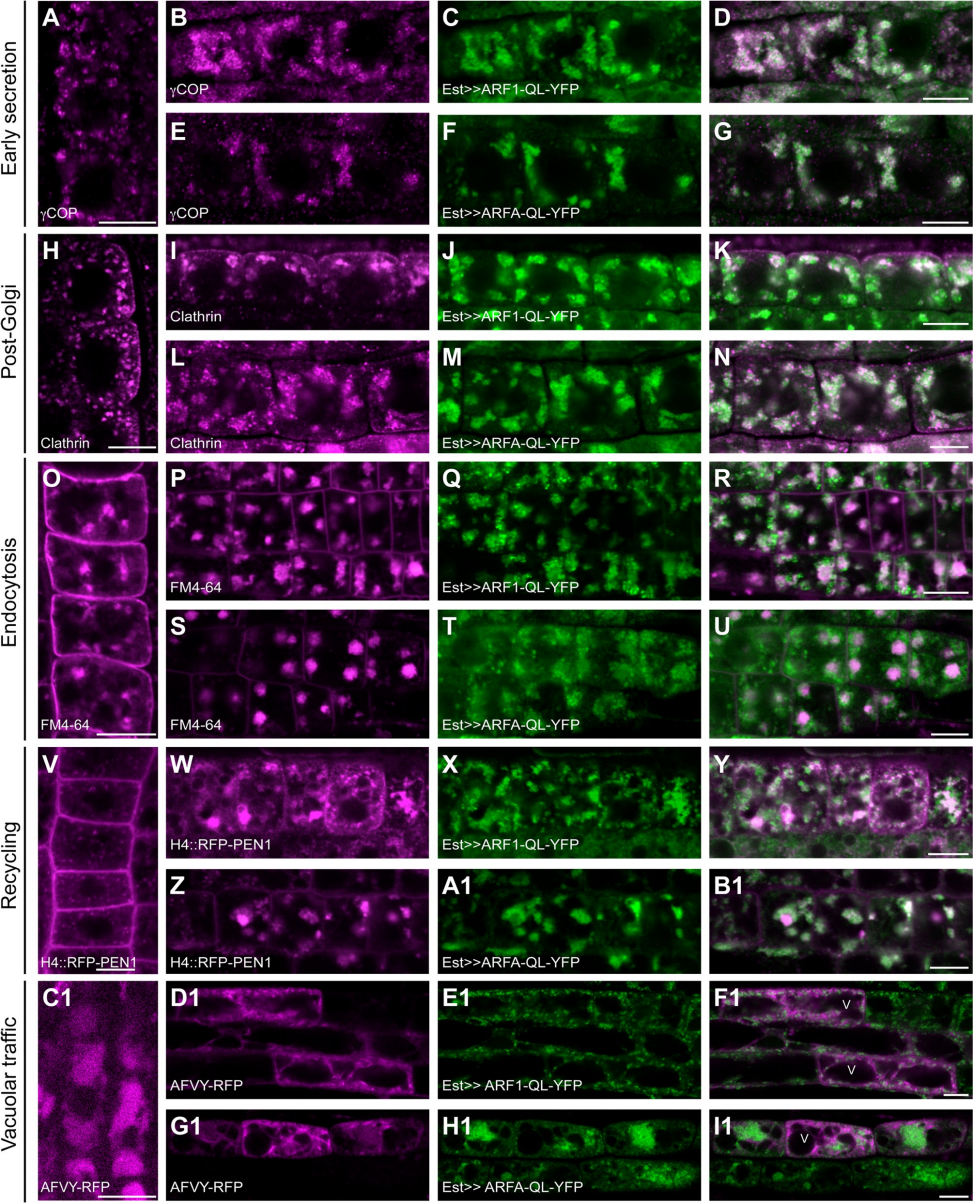
Hydrolysis-impaired forms of ARF1 and ARFA block early secretion, endosomal recycling as well as post-Golgi and vacuolar trafficking. Expression of YFP-tagged hydrolysis-impaired Q71L (QL; green) variants of ARF1 and ARFA was induced by 20μM estradiol for 5-6h and trafficking markers (magenta) were analyzed in immunostaining (A-N) or live-cell imaging (O-I1). (**A-G**) The COPI subunit γCOP (A, B, E) was recruited to the Golgi in wild-type control (A; Col). Expression of ARF1-QL-YFP (C) and ARFA-QL-YFP (F) induced aggregation of γCOP (B, E) co-localizing with ARF1 and ARFA (D, G). (**H-N**) Clathrin coat (H, I, L) was recruited to the TGN in wild-type control (H). In contrast, clathrin formed aggregates and co-localized with ARF1-QL-YFP (I-K) and ARFA-QL-YFP (L-N). (**O-U**) BFA treatment (50μM for 1h) was used to visualize endocytosed FM4-64 (O, P, S) in BFA-compartments. Endocytosis of FM4-64 in ARF1-QL-YFP (P-R) and ARFA-QL-YFP (S-U) was comparable to wild-type control (O). (**V-B1**) Seedlings were treated with 20μM estradiol and 50μM BFA for 5h. Recycling of H4::RFP-PEN1 was analyzed after BFA wash-out for 2h. H4::RFP-PEN1 (V, W, Z) returned to the plasma membrane in wild-type control (V). ARF1-QL-YFP (W-Y) and ARF A-QL-YFP (Z-B1) interfered with recycling. (**C1-I1**) Trafficking of the vacuolar cargo, AFVY-RFP (C1, D1, G1), expressed from the estradiol-inducible system, was inhibited in ARF1-QL-YFP (D1-F1) and in ARFA-QL-YFP (G1-I1) expressing lines but not in wild-type (C1). Scale bar, 10μm. The same wild-type controls were used as in Figs 3 and S3 and S5.

The hydrolysis-impaired forms ARF1-Q71L-YFP and ARFA-Q71L-YFP both influenced ARF1-dependent trafficking pathways. This was shown by using specific markers, which also revealed different effects between the two Q71L forms and ARF1-T31N-YFP (Fig 5, compare with Fig 3). This disparity was further reflected by differences in ultrastructural abnormalities between ARF1-T31N-YFP on one hand and ARF1-Q71L-YFP or ARFA-Q71L-YFP on the other, with the latter resulting in the budding of large vesicles from the Golgi stacks (Fig 4F, 4G and 4K–4M). Coat proteins γCOP and clathrin stayed on the membrane in large aggregates in cells expressing both ARF-Q71L-YFP variants, in contrast to their cytosolic retention in cells expressing ARF1-T31N-YFP (Fig 5A–5N, compare with Fig 3A–3D and 3H–3K). There was no adverse effect on ER morphology, unlike in ARF1-T31N-YFP (S7A–S7G Fig; compare with S3R–S3U Fig). However, the two Q71L ARFs blocked KNOLLE trafficking to the cell division plane (S7H–S7O Fig). Similarly, there was only a slight but noticeable effect on the secretion of SYP132 and xyloglucan (S7P–S7V, S6M–S6R and S6V–S6X Figs). There was no obvious effect on endocytosis of FM4-64 (Fig 5O–5U) or Qa-SNARE PEN1 (S7W–S7C1 Fig). An earlier study reported no endocytic uptake of FM4-64 after 3h heat-shock induction of ARF1-QL [20]. The reason for the difference between their results and ours is not clear at present but might be related to different experimental conditions.

Recycling of PEN1 to the plasma membrane appeared to be blocked in ARF1-Q71L-YFP and ARFA-Q71L-YFP root cells (Fig 5V–5B1). In addition, vacuolar trafficking of AFVY-RFP was impaired, resulting in its accumulation in endosomal compartments (Fig 5C1–5I1). In conclusion, both ARFA-Q71L-YFP and ARF1-Q71L-YFP appeared to interfere with ARF1-dependent trafficking, possibly by depleting necessary effector proteins.

### Interaction of ARF1 and ARFA with ARF-GEFs assayed by co-immunoprecipitation

**Fig 6:**
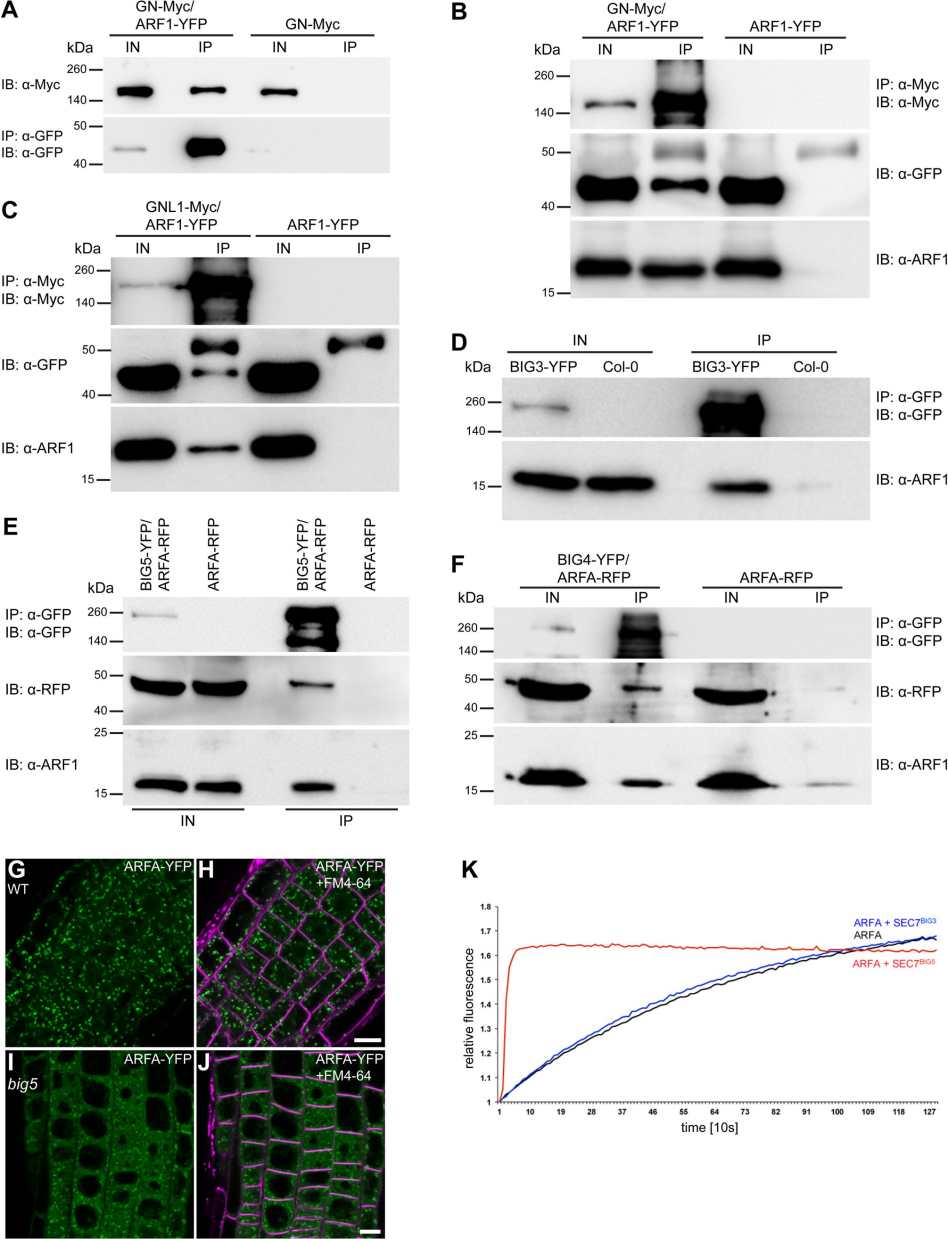
Interaction of ARF1 and ARFA with different ARF-GEFs and localization of ARFA in *big5* mutant. (**A-F**) Co-immunoprecipitation (Co-IP) studies. (**A**) Co-IP of GNOM-Myc with ARF1-YFP, using GFP-Trap-agarose beads (IP: α-GFP) followed by immunoblot analysis (IB) with α-Myc antibody. Seedlings expressing only GNOM-Myc (GN-Myc) were used as control. (**B**) Co-IP of endogenous and YFP-tagged ARF1 with GNOM-Myc, using α-Myc-agarose beads (IP) followed by IB with α-GFP antibody and α-ARF1 antibody to detect YFP-tagged and endogenous ARF1. (**C**) Co-IP of endogenous and YFP-tagged ARF1 with GNL1-Myc, using α-Myc-agarose beads (IP) followed by IB analysis with α-GFP antibody and α-ARF1 antibody. (**D**) Co-IP of endogenous ARF1 with BIG3-YFP, using GFP-Trap-agarose beads (IP: α-GFP) followed by IB with α-ARF1 antibody. Col, wild-type control. (**E**) Co-IP of ARFA (ARFB1c)-RFP and endogenous ARF1 with BIG5-YFP, using GFP-Trap-agarose beads (IP: α-GFP) followed by IB analysis with α-RFP antibody and α-ARF1 antibody. (**F**) Co-IP of ARFA (ARFB1c)-RFP and endogenous ARF1 with BIG4-YFP, using GFP-Trap-agarose beads (IP: α-GFP) followed by IB analysis using α-RFP antibody and α-ARF1 antibody. IN, input; IP; immunoprecipitate; IB, immunoblot; kDa, kilodalton. (**G-J**) ARFA-YFP localization in wild-type (WT) (G-H) and *big5* mutant (I-J). Note the highly cytosolic signal of ARFA-YFP in *big5*. Scale bars, 10μm. (**K**) In-vitro GDP-GTP exchange activity of the catalytic SEC7 domain of BIG3 (blue) and BIG5 (red) on ARFA. Negative control, ARFA alone (black). See also S5 Data.

Considering the differential effects of ARF1 and ARFA on trafficking pathways, we examined the interaction between the two ARF GTPases and ARF-GEFs by co-immunoprecipitation with protein extracts from seedlings expressing differently tagged ARFs and ARF-GEFs. Co-IPs in both directions yielded comparable results (Figs 6A–6F and S8A–S8J). The interaction between ARF1 and the BFA-sensitive ARF-GEF GNOM was enhanced by BFA treatment of seedling roots (Figs 6A and 6B and S8B). ARF-GEFs GNOM, GNL1, GN::GNL2 (GNL2 is normally only expressed in pollen but not in seedlings; [13]), BIG3, BIG4 and BIG5 all co-immunoprecipitated ARF1 (Figs 6A–6F and S8B–S8J). Unlike ARF1, ARFA was not co-immunoprecipitated with GNOM (S8E and S8F Fig) or GNL1 (S8G Fig). Thus, ARF1 interacted with all known ARF-GEFs whereas ARFA did not interact with ARF-GEFs in retrograde Golgi-ER traffic and in GNOM-dependent endosomal recycling to the basal plasma membrane.

ARF1-YFP and ARFA-YFP interacted with BIG5 as revealed by co-IP (Figs 6E and S8D). Since the interaction between ARFs and ARF-GEFs takes place on membranes we analyzed the subcellular localization of ARFA in *big5* mutant seedlings, which revealed highly cytosolic localization of ARFA-YFP (Figs 6G–6J and S8K–S8N). Furthermore, co-IP also detected weak interaction between BIG4-YFP and ARFA-RFP (Fig 6F). However, no interaction was detected between ARFA-RFP and BIG3-YFP (S8H Fig). Thus, ARFA might be activated by both BIG5 and a subset of the late-secretory ARF-GEFs BIG1-4.

GDP-GTP exchange on ARF proteins is catalyzed by the SEC7 domain of ARF-GEFs [34, 35]. We have previously shown that GNOM has exchange activity on mammalian ARF1 in vitro in a BFA-sensitive manner [36]. Also, the recombinant SEC7 domain of BIG3 is able to catalyze GDP-GTP exchange of *Arabidopsis* ARF1 in vitro in a BFA-resistant manner [37, 38]. However, the same SEC7 domain of BIG3 did not significantly stimulate GDP-GTP exchange of TGN-localized ARFA (aka ARF8) or ARFB (aka ARF9) in vitro [38]. We also tested the in-vitro exchange activity of the recombinant SEC7 domain of TGN-localized BIG5. BIG5 (aka MIN7 and BEN1) appears to play roles in pathogen response and endocytic trafficking [15–18]. In contrast to BIG3, BIG5 catalyzed the GDP-GTP exchange on ARFA (Fig 6K). Because BIG5 also interacted with ARF1 in planta, the SEC7 domain of BIG5 appears to have dual ARF substrate specificity.

To confirm that the interaction of ARFA with BIG5 reflects a substrate-enzyme relationship, we performed co-immunoprecipitation of BIG5-RFP with ARFA-T31N-YFP and ARFA-Q71L-YFP, respectively, and used co-immunoprecipitation of GNOM with ARF1-T31N-YFP or ARF1-Q71L-YFP as controls. In both cases, the ARF-GEFs only interacted with the activation-impaired T31N form, indicating that the ARFs are used as substrates (S8I–S8J Fig). We then directly tested by in-vitro GDP-GTP exchange assay whether ARFA(wt) and the mutant forms ARFA-T31N and ARFA-Q71L are substrates of BIG5. We also used ARF1(wt) and ARF1-T31N as controls. The SEC7 domain of BIG5 catalyzed GDP-GTP exchange on ARF1(wt), ARFA(wt) and ARFA-Q71L but not on ARF1-T31N nor on ARFA-T31N (S9 Fig). ARFA appeared to be activated faster than ARF1. In addition, ARFA-Q71L reached a seemingly higher level of activation than ARFA(wt), presumably because GTPγS dissociated more slowly. In contrast, both ARF1-T31N and ARFA-T31N did not show any change over time, resembling the GDP-GTP exchange reaction in the absence of GTPγS (S9 Fig). In conclusion, ARFA-T31N interfered with GDP-GTP exchange essentially like ARF1-T31N and thus, a reason for the lack of biological effect of ARFA-T31N in contrast to ARF1-T31N has to be sought elsewhere (see Discussion).

Of the four functionally redundant late secretory ARF-GEFs BIG1 to BIG4, BIG3 interacted with ARF1 but not with ARFA whereas BIG4 interacted with both ARF1 and ARFA (see Figs 6F and S8H). This observation could in principle explain why strong ARFA-T31N expression had no deleterious effect. To assess the dual-specificity of ARF-GEF BIG4 in planta, we strongly expressed Est>>ARFA-T31N-YFP in a *big3 UBQ10*::*BIG4*^*R*^-YFP (engineered BFA-resistant BIG4 [14]) background. This genetic background in conjunction with brefeldin A (BFA) treatment leaves only BIG4 active whereas the functionally overlapping ARF-GEFs BIG1 and BIG2 are inhibited and BIG3 is genetically knocked out. Combined EST and BFA treatment of seedlings resulted in approximately 30% reduction of root growth, suggesting that ARFA-T31N-YFP interfered with the activation of ARF1 in the late secretory pathway required for primary root growth (S4L Fig).

### The catalytic SEC7 domain did not confer specificity of action of ARF1-activating ARF-GEFs

**Fig 7:**
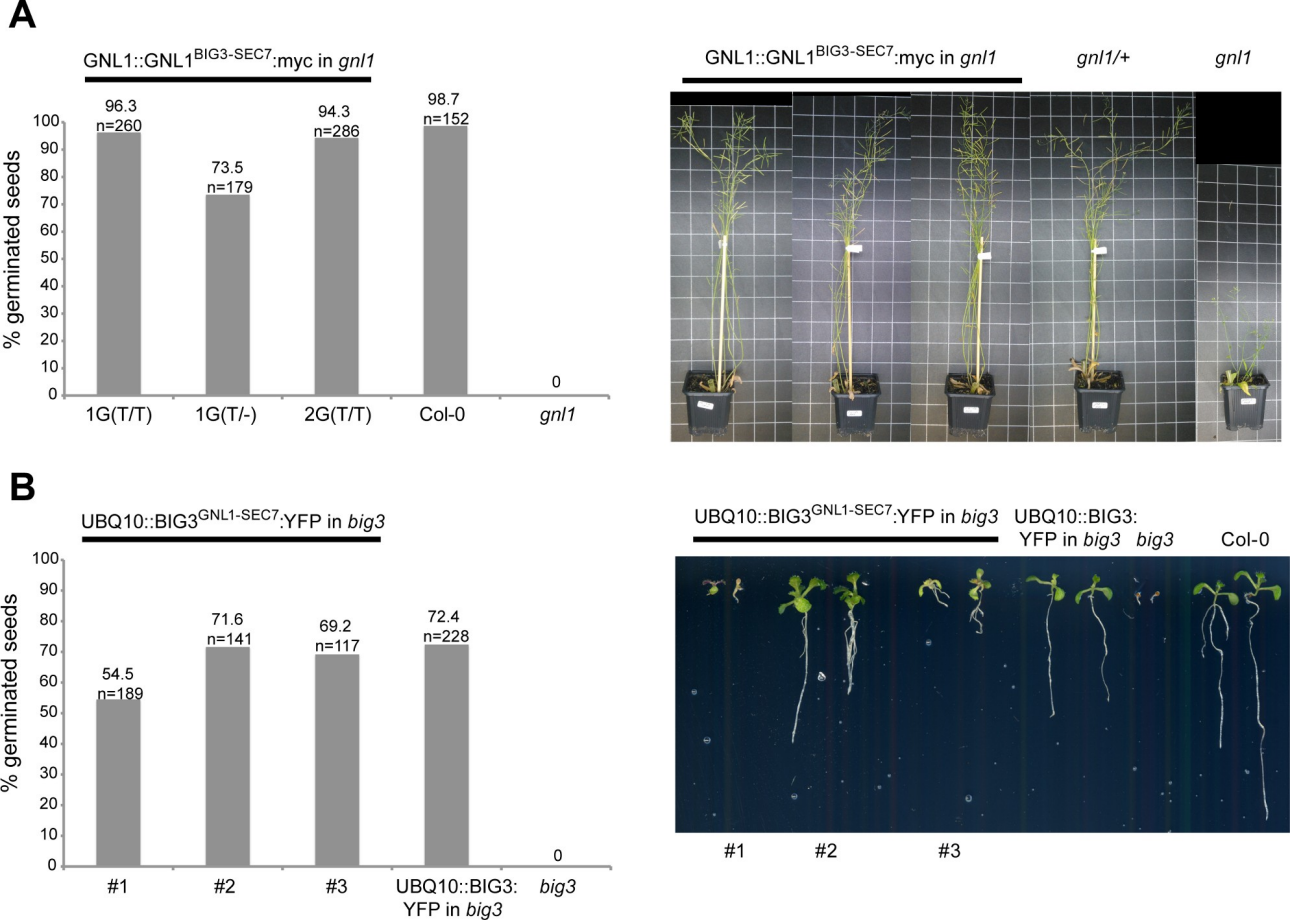
Swapping of catalytic domains between Golgi-localized and TGN-localized ARF-GEFs did not impair ARF-GEF function. **(A)** *Left panel*: Expression of *GNL1*^*SEC7(BIG3)*^:*myc* transgene complements the germination defect of *gnl1* mutant seeds on agar plates containing 7μM BFA. Col-0, wild-type control; T/T, homozygous for transgene; T/-, hemizygous for transgene. *Right panel*: Expression of *GNL1*^*SEC7(BIG3)*^:*myc* transgene rescues the stunted growth phenotype of *gnl1* mutant plants. **(B)** *Left panel*: Expression of *BIG3*^*SEC7(GNL1)*^:*YFP* transgene complements the germination defect of *big3* mutant seeds on agar plates containing 5μM BFA. All *BIG3*^*SEC7(GNL1)*^:*YFP* lines are hemizygous for the transgene. *UBQ10*::*BIG3*:*YFP*, positive control. *Right panel*: Phenotype of *big3* mutant seedlings rescued by expression of *BIG3*^*SEC7(GNL1)*^:*YFP* transgene compared to *big3* mutant, wild-type (Col-0) control, and *BIG3*:*YFP* transgenic seedlings (positive control); on agar plates containing 5μM BFA. See also S6 Data.

The interaction assays suggested that ARF1 is a common substrate of all ARF-GEFs in *Arabidopsis*. If this is the case, the SEC7 domain might be swapped between ARF-GEFs without affecting the activity of the respective ARF-GEFs. We generated GNL1-BIG3 chimeras with swapped SEC7 domains. In two transgenic lines tested, cis-Golgi-localized GNL1 with the SEC7 domain of the TGN-localized ARF-GEF BIG3 in place of its own was able to rescue *gnl1* mutant plants, thus complementing the BFA-sensitive seed germination phenotype and the post-embryonic stunted growth of *gnl1* knockout mutant plants (Fig 7A). In three transgenic lines tested, BIG3 with the SEC7 domain of GNL1 in place of its own complemented BFA-sensitive seed germination of *big3* knockout mutant and rescued the seed germination defect of *big3* mutant seedlings to nearly the same extent as did the parental *BIG3* transgene (Fig 7B). In conclusion, the SEC7 domain did not confer specificity of action to ARF1-activating ARF-GEFs.

**Fig 8:**
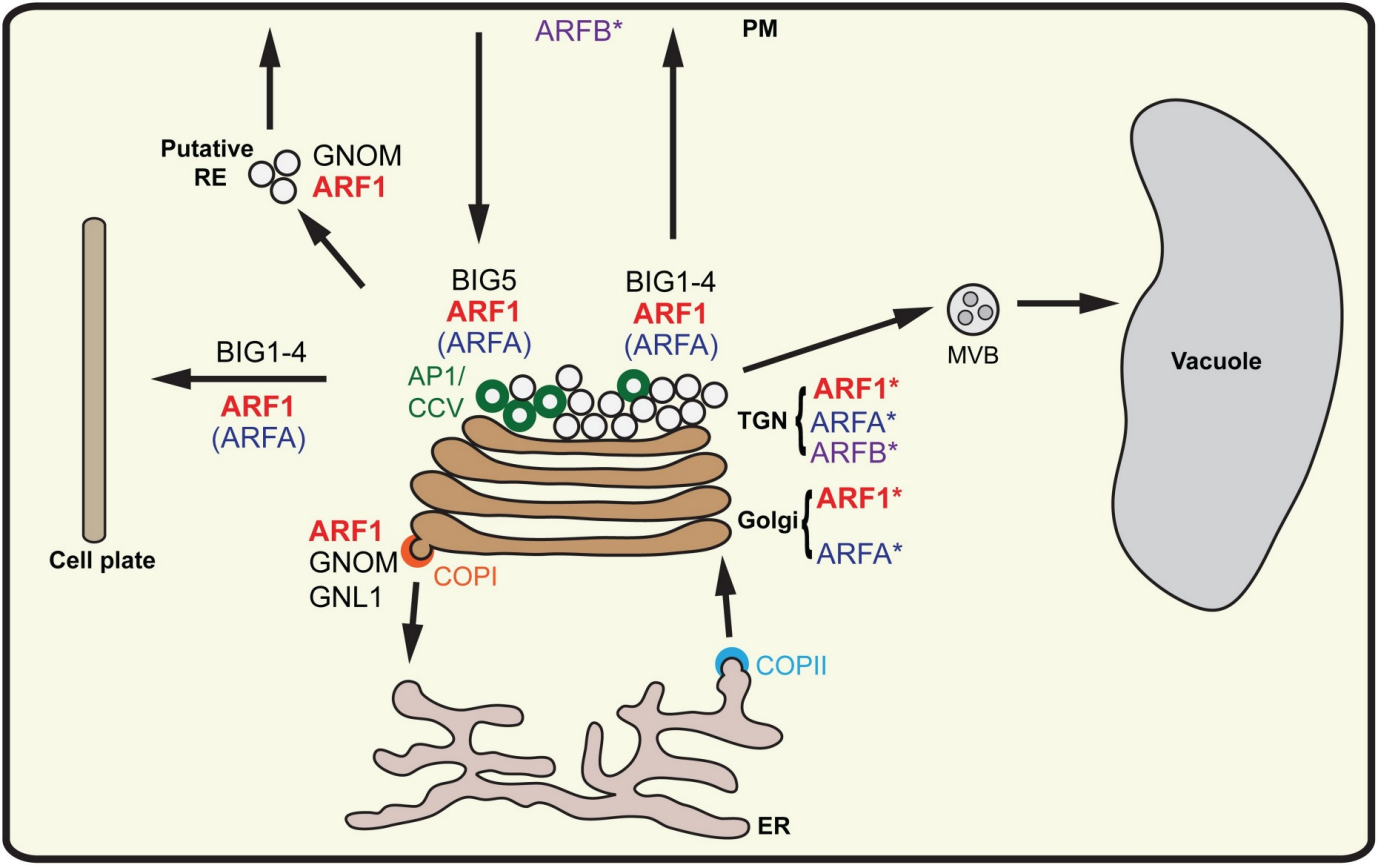
Interactions between ARFs and ARF-GEFs in various trafficking pathways in *Arabidopsis* (model). Class-I ARFs (ARF1) are recruited to Golgi stack and TGN. Class-A ARFs (ARFA) are mainly present at TGN, a minor fraction localizing at the Golgi stack. Class-B ARF (ARFB) localized at PM and at TGN. GNOM and GNL1 activate class-I ARFs (ARF1) at the Golgi to carry out COPI-mediated Golgi-ER retrograde trafficking. Activation of class-I ARFs (ARF1) at the TGN by BIG1-4 occurs in secretory pathways to PM, cell plate and vacuole. ARF1 is also activated by BIG5 during endocytosis and by GNOM during recycling to the PM. In addition to ARF1, BIG5 and a subset of BIG1-4 could also activate ARFA (and possibly, ARFB), which plays no essential role in any trafficking pathway. AP1/CCV, adaptor protein complex 1/clathrin-coated vesicles; MVB, multivesicular body; PM, plasma membrane; RE, recycling endosomes; TGN, trans-Golgi network. The asterisk indicates localization of individual ARF GTPases whereas unmarked ARFs represent probable functional involvement in the respective transport steps.

## Discussion

Our results strongly suggest that, in contrast to mammals and yeast, *Arabidopsis* can successfully carry out essential membrane trafficking with only one class of ARF GTPase, represented by isoforms of ARF1, and that this single class of ARF GTPase can be activated by all eight ARF-GEFs acting in different trafficking pathways–secretion (including cytokinesis and vacuolar traffic), endocytosis, and recycling (Fig 8). There are no orthologs of non-plant class-II and class-III ARFs in *Arabidopsis* or other flowering plants whereas the putative ARFs of the plant-specific classes A and B appear not to be essential. The single ARFB localized mainly to the plasma membrane and has therefore been compared to mammalian Arf6 [24]. However, neither *amiR(ARFB)* nor ARFB-T31N overexpression caused any mutant phenotype and ARFB-Q71L overexpression also had no deleterious effect. Thus, ARFB plays no essential role or its role might be taken over by some functionally overlapping ARF protein. To test for that possibility, we generated estradiol-inducible *ARFA-T31N ARFB-T31N* co-overexpression plants as well as plants lacking *ARFA* and *ARFB* transcripts. Again, there was no deleterious effect. Thus, by whatever criteria used, ARFB protein appears to be of no vital importance. This would be consistent with the phylogenetic analysis indicating that ARFB only arose late in dicot angiosperm evolution and is not present in the vast majority of dicots nor in any of the monocots whose genome has been analyzed, suggesting a specialized role not essential for the majority of trafficking processes. Alternatively, ARFB might not be an ARF GTPase but an ARF-related protein acting in a trafficking-unrelated process. In contrast to ARFA-Q71L, ARFB-Q71L did not interfere with ARF1 trafficking, although by sequence, ARFB is more closely related to ARF1 than is ARFA.

Unlike ARFB, ARFA is evolutionarily conserved in the plant lineage. However, the double mutant *of arfA (B1b B1c)* did not show any obvious mutant phenotype and overexpression of ARFA-T31N had no deleterious effect. Thus, ARFA was not required for any essential trafficking pathway. The lack of any deleterious biological effect of ARFA-T31N raised doubts about the functionality of this mutant form of ARFA because one might presume that it ought to block the activation of ARFA and ARF1 if one or more ARF-GEFs activated both ARFA and ARF1 and if ARFA-T31N behaved like ARF1-T31N in its interaction with the SEC7 domain of ARF-GEFs. The in-vitro GDP-GTP exchange assays demonstrated that ARFA-T31N is not activated by the SEC7 domain of BIG5, although co-immunoprecipitation revealed interaction of ARF-GEF BIG5 with ARFA-T31N but not with ARFA-Q71L. Interestingly, ARFA-Q71L interfered with ARF1-dependent trafficking, suggesting depletion of coat proteins and/or other effectors that are also recruited by ARF1. ARFA-T31N overexpression blocked the deleterious effect of ARFA-Q71L but, unlike ARF1-T31N, had no deleterious effect on its own. Moreover, T31N acted like an intragenic suppressor of ARFA-Q71L in the ARFA-T31N,Q71L double mutant, and overexpression of this double mutant also blocked the deleterious effects of ARFA-Q71L. Furthermore, strong overexpression of ARF1 was also able to suppress the inhibitory effect of ARFA-Q71L on secretion. Thus, ARFA appears to be activated by some endogenous ARF-GEF(s) that also activate(s) ARF1 but is/are not essential for activating ARF1. Indeed, ARFA interacted with BIG5 and BIG4, both of which also interacted with ARF1. BIG5 seems to be not essential for plant viability [17, 18], and BIG4 acts redundantly with BIG1, BIG2 and BIG3 in the late secretory pathway [14]. However, the ARF-GEF BIG3 did not interact with ARFA and its SEC7 domain did not activate ARFA but only ARF1 [38], which would explain why ARFA-T31N cannot block the essential late-secretory pathway to the plasma membrane in interphase and to the cell-division plane in cytokinesis [14]. This conclusion was experimentally supported by demonstrating that ARFA-T31N-YFP expression impaired root growth when only BIG4 of the four functionally redundant late-secretory ARF-GEFs BIG1 to BIG4 was functional. The essential early-secretory pathway between ER and Golgi is most likely not affected by ARFA-T31N because ARF-GEF GNOM, and probably also its paralog GNL1, does not interact with ARFA. If ARFA is not essential why has it been conserved in plant evolution? A possible scenario might be that ARFA mediates a trafficking pathway that is non-essential under laboratory conditions because of overlap with a parallel ARF1-dependent trafficking pathway but might become limiting, for example, in fluctuating environmental conditions.

Our results strongly suggest that ARF1 is the only functionally required ARF GTPase to mediate all fundamental trafficking pathways (except SAR1-dependent COPII trafficking for which it is only indirectly required) in *Arabidopsis* such that blocking ARF1 function essentially corresponds to knocking out all ARF-GEFs (Fig 8). Strong expression of ARF1-T31N from fertilization onward arrested embryo development very early, resulting in a multi-nucleate cell, which corresponds to shutting down all membrane trafficking pathways. To break down the global requirement of ARF1 into specific trafficking pathways, we studied the effects of inducible ARF1-T31N overexpression on the subcellular localization of relevant trafficking markers and compared the effects with those of inducibly knocking out specific ARF-GEFs for which we also demonstrated interaction with ARF1 in planta. One result was that ARF1 together with ARF-GEFs GNL1 and GNOM mediates COPI-dependent retrograde Golgi-ER traffic whereas ARF1 together with ARF-GEFs BIG1-4 mediates post-Golgi membrane trafficking from the TGN to the plasma membrane, the vacuole and the cell-division plane. As a consequence, we hypothesized that if indeed ARF1 was the common substrate of all ARF-GEFs regardless of the trafficking pathway, their catalytic SEC7 domains might be interchangeable. We demonstrated this by swapping the SEC7 domains between GNL1 and BIG3, which gave chimeric proteins that were able to rescue the respective mutants to the same extent as did the parental proteins. Thus, by all available criteria, ARF1 appears to be the only class of ARF GTPase required for the diverse trafficking pathways of flowering plants. In contrast, the ARF-GEFs mediating specific pathways cannot replace each other, suggesting that they might play specific roles in traffic regulation beyond the activation of ARF1 by GDP-GTP exchange and that these functions are exerted by the non-catalytic domains.

## Materials and methods

### Plant material and growth conditions

*Arabidopsis thaliana* plants were grown on soil or agar plates in growth chambers under continuous light conditions at 23°C. *arfB1b* mutant line was ordered from INRA (Flag_289D05; http://www-ijpb.versailles.inra.fr/) and *arfB1c* mutant line from GABI-KAT (GABI_526B04; http://www.gabi-kat.de). *arfB1b* mutant line was selected on MS plates using Kanamycin; *arfB1c* mutant line was selected on MS plates using Sulfonamide. The *big5* T-DNA line has been described earlier (AtMIN7 KO #3 [15]).

Previously published lines that were used in this study:

VHA-a1-RFP [39], Wave22-mCherry [40], H4::RFP-PEN1 [41], Est>>AFVY-RFP [14], N-ST-YFP [42], sec-mCherry-HDEL [32], YFP-RABG3f [40, 43], GNOM-Myc [11], GNL1-Myc [12], GNOM::GNL2-GFP [13], UBQ10::BIG4-YFP [14], BIG3::BIG3-YFP [14].

*Nicotiana tabacum* L. SR1 plants were grown on MS medium supplemented with 2% sucrose, 0.5 g l^–1^ MES and 0.8% Agar at pH 5.7 in 16/8 h day-night cycles at 22°C.

### Genotyping of *arf* T-DNA insertion lines

Primers for testing *arfB1b* heterozygous mutant:
5‘AGGTCTCAGTTTCTCTTGGCC 3 ’and
5‘CGTGTGCCAGGTGCCCACGGAATAGT 3 ’
Testing for *arfB1b* homozygous mutants:
5‘AGGTCTCAGTTTCTCTTGGCC 3 ’and 5‘TTTGTAAATTGGGCTGCAAAG 3
Primers for testing *arfB1c* heterozygous mutant:
5‘AGGGAACTTGAATCTTCAGGG 3’and 5‘ACGAGTCGAGAAACGATGTTG 3’
Testing for *arfB1c* homozygous mutant:
5‘AGGGAACTTGAATCTTCAGGG 3’and 5‘ATATTGACCATCATACTCATTGC 3 ’

### Generating transgenic plants

CDS of *ARF1* (*ARFA1c)*, *ARFB* (*ARFB1b* and/ or *ARFB1c)* and *ARFB* (*ARFB1a)* were amplified using Sense-Primers containing *Apa*I restriction site and Antisense-Primers containing *Avr*II and *BamH*I restriction sites. Amplified ARF fragments were introduced in pGreen (Hyg)-RPS5A-tNos cassette via *Apa*I and *BamH*I restriction sites [31]. C-terminal YFP tag was inserted via *Avr*II restriction site.

Site-directed mutagenesis using the following primers was performed to introduce T31N or Q71L mutations:

*ARFA1c-T31N*: 5‘CTTGTAGAGGATAGTATTCTTACCAGCAGCATC 3’
*ARFB1a-T31N*: 5‘CTTGTAAAGGATGGTATTCTTCCCTGAACCATC 3’
*ARF B1b-T31N*: 5‘CTTGTAGAGAATAGTATTTTTTCCAGCAGCATC 3’
*ARFA1c-Q71L*: 5‘GGACGGATCTTGTCTAGACCCCCAACATCCC3’
*ARFB1a-Q71L*: 5‘CAATTTGCGAATCTTCTCTAGTCCTCCTATATCCC 3’
*ARFB1b-Q71L*: 5‘GAGGTCTCAGTTTCTCTAGGCCACCAACATCCC 3’

*ARF-T31N-YFP* and *ARF-Q71L-YFP* were amplified and cloned into pDONR221 (Invitrogen) generating a pEntry clone. Afterwards, LR reaction was performed introducing *ARF-T31N-YFP* and *ARF-Q71L-YFP* into a modified β-estradiol inducible pMDC7 vector [44] in which Ubiquitin 10 promoter replaced the original promoter (kindly provided by Niko Geldner, Univ. Lausanne).

*ARFA(B1b)-TN*, *QL-YFP* construct was generated by site-directed mutagenesis on *pENTRY-ARFA-TN-YFP* using the following primer to introduce Q71L mutation:

*ARFB1b-Q71L*: 5‘GAGGTCTCAGTTTCTCTAGGCCACCAACATCCC 3’.

Afterwards, LR reaction was performed introducing *ARFA-T31N*, *Q71L-YFP* into the modified β-estradiol inducible pMDC7 vector [44] including Ubiquitin 10 promoter (see above).

T1 plants of RPS5A::ARF-YFP, Est>>ARF-TN-YFP, Est>>ARF-QL-YFP, Est>>ARFA-TN, QL-YFP were selected with hygromycin.

Artificial micro RNA of *ARFB1a* was cloned according to Artificial microRNA Designer: (http://wmd3.weigelworld.org/cgi-bin/webapp.cgi; [45]) using the following primers:

B1a_miR-s_I: 5´ gaTTCGTACGAATCAGAGCGTACtctctcttttgtattcc 3’
B1a_miR-a_II: 5‘gaGTACGCTCTGATTCGTACGAAtcaaagagaatcaatga 3’
B1a_miR*s_III: 5‘gaGTCCGCTCTGATTGGTACGATtcacaggtcgtgatatg 3’
B1a_miR*a_IV: 5‘gaATCGTACCAATCAGAGCGGACtctacatatatattcct 3

The amiRNA precursor fragment was cloned into pGII (Bar)-RPS5A-tNos expression cassette [31]. T1 plants were selected using Phosphinotricine. UBQ10::BIG5-YFP was cloned by amplifying BIG5 CDS from a cDNA library, cloned into pDONR221 (Invitrogen) and afterwards into UBQ10::YFP destination vector [46]. T1 plants were selected by using phosphinotricine.

Generation of SEC7 Swap constructs:

PCRs to generate a BIG3-SEC7 fragment with GNL1 sequence overlaps at the borders were performed with the following primers:

GNL1_BIG3-SEC7 upstream sense: 5’ GCAATGATGGCGCCTTATGGTATAC 3’
GNL1_BIG3-SEC7 upstream anti:
5’ ctaaggcatcagaattcccacttccgtaattctcacatctcac 3’
GNL1_BIG3-SEC7 downstream sense:
5’ gatgatggtttgggtccgcagcaactgatgacagccagtcg 3’
GNL1_BIG3-SEC7 downstream anti: 5’ GCTCTTATAAGAGAGTTAACAAGC 3’
GNL1_BIG3-SEC7 sense:
5’ gtgagatgtgagaattacggaagtgggaattctgatgccttag 3’
GNL1_BIG3-SEC7 anti:
5’ cgactggctgtcatcagttgctgcggacccaaaccatcatc 3’
The chimeric fragment was introduced into a pGII (Hyg) GNL1::GNL1:myc backbone with EheI and HpaI restriction enzymes.
PCRs to generate a GNL1-SEC7 fragment with BIG3 sequence overlaps at the borders were performed with the following primers:
BIG3_GNL1-SEC7 upstream sense: 5’ GTTCTTAGGCGCCATCAAGCAG 3’
BIG3_GNL1-SEC7 upstream anti:
5’ gggaacccaaaaattaggatcggacaattcagactgagagtc 3’
BIG3_GNL1-SEC7 downstream sense:
5’ ggacgaagataaaggaaccggcttgcagaagcagcctacaaact 3’
BIG3_GNL1-SEC7 downstream anti: 5’ GTAAATATCCGACTCATGTCACCAAC 3’
BIG3_GNL1-SEC7 sense:
5’ gactctcagtctgaattgtccgatcctaatttttgggttccc 3’
BIG3_GNL1-SEC7 anti:
5’ agtttgtaggctgcttctgcaagccggttcctttatcttcgtcc 3’
The chimeric fragment was introduced into a pGII (BAR) UBQ10::BIG3:YFP backbone with EheI and BoxI restriction enzymes.
Generation of *UBQ10*::*BIG5-YFP* and *UBQ10*::*BIG5*^*R*^*-RFP*:
BIG5 CDS was amplified with
BIG5-sense:
5’ AAAAAGCAGGCTGGATGGCGGCTGGTGGATTTTTGACTC 3’
BIG5-antisense:
5’ AGAAAGCTGGGTCCTGTTGCAAAAGTGGCTTCAATTG 3’
and cloned into pDONR221 (Invitrogen) generating a pEntry clone.
Amplified BIG5 CDS was a non-annotated splice variant. Thus, the 12^th^ Intron was cloned into pENTRY-BIG5.
Site directed mutagenesis was used to introduce Tyrosine^727^ to Phenylalanine and Methionine^712^ to Leucin mutations generating BFA-resistant version of BIG5 (BIG5^R^).
5’ CTGTATTTAACAgGATAACTGCAaAGGCTAGAACGTAGGC 3’
LR reaction was performed introducing BIG5 into pUBC-YFP/RFP-DEST destination vector containing C-terminal YFP or RFP [46].

### Cloning of constructs for transient expression in protoplasts

Expression cassette generation: The vector pFF04 [47] was modified to contain a *mas* promoter sequence, *NLS-GFP*, and a 35S terminator sequence. The modified vector was called pFK059. The *mas* promoter is bidirectional, regulating transcription of the gene of interest on one side and of *NLS-GFP* on the other side. The latter can be used as an indirect expression control. The sequences were PCR amplified to introduce restriction sites for cloning with the following primers:

*mas* promoter ClaI sense: 5’ AGTCTAATCGATTTGGTGTATCGAG 3’
*mas* promoter NheI anti: 5’ AGTCTAGCTAGCACGTGTGGAAGATATG 3’
NLS-GFP ClaI sense:
5’ AGTCTAATCGATGGCTCCAAAGAAGAAGAGAAAGGTCATGGTGAGCAAGGGCGA 3’
GFP Stop PstI anti: 5’ AGTCTACTGCAGCTACTTGTACAGCTCGTCC 3’
35S terminator PstI sense: 5’ CTGCAGGTCCGCAAAAATCACCAGTCTCTCTCT 3’
35S terminator EcoRI anti: 5’ GAATTCGAATCATACATAAACATAAACATT 3’
CDS of *ARFA1c* and *ARFB1b* were amplified using Sense-Primers containing *NheI* restriction site and Antisense-Primers containing BamHI restriction site. Amplified *ARF* fragments were introduced in pFK059 via NheI and BamHI restriction sites.
ARFA1c NheI sense: 5’ GATCTCGCTAGCATGGGGTTGTCATTCGGAAAGTT 3’
ARFA1c BamHI anti: 5’ GGCAGTGGATCCCTATGCCTTGCTTGCGATGTTGT 3’
ARFB1b NheI sense: 5’ ATAGCTAGCATGGGTCAAGCTTTTCGTAAG 3’
ARFB1b BamHI anti: 5’ ATTGGATCCTTAAAACGAGTGGCCAACC 3’
CDS of *ARFA (B1b)-TN* and *ARFA (B1b)-QL* (see “Generating transgenic plants” section) were amplified using the corresponding NheI/BamHI primers and introduced into pFK059.
A fragment from pFK059-ARFA-TN containing T31N mutation was cloned into pFK059-ARFA (B1b)-QL via AccI and NheI restriction sites to create pFK059-ARFA (B1b)-TN,QL.

### RNA extraction and RT-PCR

RNA was isolated using peqGOLD RNA extraction kit (VWR) and cDNA was generated from 1μg RNA using Thermo Scientific *Maxima H* Minus Reverse Transcriptase Kit (Thermo Scientific). Complete CDS of ARF GTPases (from start to stop) were amplified using 0.5μl or 1μl of cDNA for PCR.

### Gal4>>UAS 2-component system and *Arabidopsis* embryo analysis

*RPS5A*::*Gal4* activator line was pollinated with *UAS*::*ARF 1-TN-YFP*, *UAS*::*ARF 1-QL-YFP*, *UAS*::*ARF A-TN-YFP* and *UAS*::*ARF A-QL-YFP* reporter lines. 3 or 5 days after pollination siliques were opened and ovules were mounted in chloral hydrate solution [48]. Images were taken at the Zeiss Axiophot using a 40x Objective.

### Immunofluorescence localization and live imaging

Five days old seedlings were incubated in 1 ml liquid growth medium (0.5x MS medium, 1% sucrose, pH 5.8) containing 20 μM Estradiol (Sigma Aldrich) for 5-6h at plant room conditions in 24-well cell-culture plates. Incubation was stopped by fixation with 4% paraformaldehyde in MTSB. Immunofluorescence staining was performed as described [49] or with an InsituPro machine (Intavis) [50].

Antibodies used: rat anti-tubulin 1:600 (Abcam), rabbit anti-AtγCOP 1:1000 (Agrisera), rabbit anti-KNOLLE 1:2000 [49], rabbit anti-clathrin (1:600) [51]. Alexa633 (Invitrogen) or Cy3-conjugated secondary antibodies (Dianova) were diluted 1:600.

Live-cell imaging was performed with 2 μM FM4-64 (Invitrogen, Molecular Probes).

Estradiol induction was performed using 20μM Estradiol for 5-6h at plant room conditions in 24-well cell-culture plates.

### Acquisition and processing of fluorescence images

Fluorescence images were acquired at the confocal laser scanning microscope TCS-SP8 from Leica or LSM880 from Zeiss, using the 63x water-immersion objectives and Leica or Zen software. Images were generated using PMT, HYD or GaAsP detectors. Airyscan detector (Zeiss) was used for images in Figs 3A–3G and 5A–5G. All images were processed with Adobe Photoshop CS3 or CS5 only for adjustment of contrast and brightness. Intensity line profile was performed with Leica software.

### Electron microscopy and correlative light electron microscopy

For thawed cryosection labeling, seedlings were fixed with 4% formaldehyde (30 min, RT) and 8% formaldehyde (90–120 min, RT), embedded in 10% gelatin and infiltrated with a mixture of 1.8 M sucrose and 20% polyvinylpyrrolidone [52]. Infiltrated gelatin blocks were mounted on stubs, frozen in liquid nitrogen and sectioned at -115°C (80 nm) in a Leica UC7/FC7 cryo-ultramicrotome. Thawed cryosections were placed on grids covered with pioloform and carbon for immunolabeling. Unspecific binding sites were blocked with PBS containing 0.2% BSA and 0.2% milk powder. Sections were labeled with rabbit anti-GFP (1:200; [53]) diluted in blocking buffer and goat anti-rabbit IgG coupled to 6 nm gold (1:30; Dianova, Hamburg) or to ultrasmall gold (Aurion, Wageningen). Gold particles were silver-enhanced using R-Gent (Aurion) for 35–40 min. Finally sections were stained with 1% aqueous uranyl acetate and embedded in a thin layer of methyl cellulose containing 0.3–0.45% uranyl acetate.

For resin section labeling with xyloglucan-specific antibodies, 1 mm long seedling root tips were high-pressure frozen (HPM 010; Bal-Tec, Balzers, Lichtenstein) in 100 μm planchettes filled with 1-hexadecene (Merck Sharp and Dohme) and freeze-substituted in acetone supplemented with 0.4% uranyl acetate and 1.6% methanol. After 50 h at -90°C, samples were warmed up to -50°C and washed 5x with acetone before they were infiltrated with 25%, 50%, 75% and 2x 100% Lowicryl HM20 at -50°C. Infiltrated samples were UV-polymerized for two days at -50°C. For immunolabeling, 70 nm thin sections were cut (Leica UC7) and mounted on coverslips for FM or slot grids covered with Pioloform for IEM and CLEM. For immunogold labeling of grids, sections were labeled as described above with mouse anti-xyloglucan antibodies (mAb CCRC-M1, 1:10; Carbosource Services, University of Georgia) and goat anti-mouse IgG coupled to 6 nm gold (1:30; Dianova, Hamburg). In some cases gold particles were silver-enhanced as described above. Resin sections were stained with 1% aqueous uranyl acetate for 4–5 min and lead citrate for 15–20 sec. For fluorescence labeling of coverslips, sections were labeled as described above with mouse anti-xyloglucan antibodies (1:10) and goat anti-mouse IgG coupled to Cy3 (1:400; Dianova, Hamburg). Resin sections were stained for DNA with 1 μg/ml Dapi (4´,6-diamidino-2-phenylindole) for 5 min and embedded in Moviol containing DABCO (1,4-diazabicyclo[2.2.2]octane) as antifading agent. Background was negligible in control experiments without first antibody. Sections were viewed using a Zeiss Axioimager M2 with a 63x/1.40 oil immersion objective. Images were taken with a sCMOS Orca-flash4.0 camera (Hamamatsu). Contrast and brightness were adapted using Photoshop software.For simultaneous double labeling with fluorescence and gold markers, sections mounted on slot grids were incubated with mouse anti-xyloglucan antibodies (1:10) as described above. Thereafter, sections were labeled with goat anti-mouse IgG coupled to 6 nm gold (60 min), directly followed by incubation with goat anti-mouse IgG coupled to Cy3 (60 min). There are enough unbound first antibodies left for fluorochrome coupled marker molecules. Slot grids were then stained with DAPI and mounted on a slide under a coverslip (in 50% glycerol) with two additional coverslips laterally placed as spacer and fluorescent images were taken (see above). Thereafter, sections were washed with double distilled water and stained with 1% aqueous uranyl acetate (5 min) and in some cases with lead citrate (15–30 sec). Stained sections were examined in a Jeol TEM (see below). Alignment and overlay of light microscopic and electron microscopic images were performed with Picture Overlay Program (Jeol).

For ultrastructural analysis, 1 mm long seedling root tips were high-pressure frozen (HPM 010) in 100 μm planchettes filled with 1-hexadecene and freeze-substituted in acetone supplemented with 2.5% osmium tetroxide (35 h at -90°C, 6 h at -60°C, 6 h at -30°C, 2 h at 0°C). Thereafter samples were washed 5x with acetone (0°C), before they were infiltrated with 10%, 25%, 50%, 75%, 2x 100% epoxy resin (Roth, Germany). Infiltrated samples were polymerized at 60°C for two days. For ultrastructural analysis, 70 nm thin sections were cut and mounted on slot grids covered with pioloform. Sections were stained with 3% uranyl acetate in 50% ethanol, followed by lead citrate and viewed in a Jeol JEM-1400plus TEM at 120 kV accelerating voltage. Images were taken with a 4K CMOS TemCam-F416 camera (TVIPS). Contrast and brightness were adapted using Photoshop software.

### Immunoprecipitation

*Arabidopsis* seedlings (5–6 days old) were ground in liquid nitrogen and suspended in lysis buffer (50mM Tris pH 7.5, 150mM NaCl, 2mM EDTA, 1% Triton X-100) supplemented with protease inhibitors (cOmplete EDTA-free, Roche). The cell lysate was cleared by centrifugation at 10,000 x g for 15 min at 4°C. The supernatant was filtered through two layers of Miracloth (Calbiochem) and incubated with α-Myc-agarose beads (Sigma) or GFP-Trap beads (Chromotek) or RFP-Trap beads (Chromotek) for binding in cold room for 2.5-3h. The agarose beads, after binding, were washed 2–4 times with wash buffer (50mM Tris pH 7.5, 150mM NaCl) containing 0.1% Triton, followed by 1–2 washes using wash buffer lacking Triton and boiled in 2x Laemmli buffer.

To perform immunoprecipitation in the presence of brefeldin A (BFA), *Arabidopsis* seedlings were treated with BFA (50 μM) for 2h before grinding. In addition, BFA (50μM) was also added to all buffers during immunoprecipitation.

For MS analysis, YFP-tagged GTPases (ARF and RABG3f) were immunoprecipitated in the presence of brefeldin A (BFA) using GFP-Trap beads (Chromotek). The beads were washed 1x with wash buffer (50mM Tris pH 7.5, 150mM NaCl) containing 0.5% Triton and 3x with wash buffer containing 0.1% Triton. Proteins bound to the beads were eluted by boiling in 2x Laemmli buffer.

To express ARF-T31N and ARF-Q71L isoforms for immunoprecipitation, 5–6 days old seedlings were incubated in liquid MS medium (0.5x MS medium, 1% sucrose, pH 5.8) containing 20 μM Estradiol (Sigma Aldrich) for 6h at plant room conditions. The seedlings were subsequently snap frozen in liquid nitrogen and stored at -80°C.

### Western blotting

Proteins were separated on SDS-PAGE, transferred to the PVDF membrane (Millipore) and detected using one of the following antibodies: α-GFP (mouse, 1:1,000, Roche), α-RFP (rat, 1:1,000, Chromotek), α-Myc (1:1,000, Sigma), α-ARF1 (rabbit, 1:2,000, Agrisera), α-GN-SEC7 (rabbit, 1:2,500) [37], and POD-conjugated secondary antibodies. Chemiluminescence signal was acquired using Fusion Fx7 detection system (PEQlab, Erlangen, Germany).

### LC MS/MS analysis

Eluted proteins were purified using SDS PAGE (Invitrogen). Coomassie-stained gel pieces were excised and in-gel digested using Trypsin as described previously [54]. Extracted peptides were desalted using C18 StageTips [55] and subjected to LC-MS/MS analysis that was performed on an Easy nano-LC (Thermo Scientific) coupled to an LTQ Orbitrap XL mass spectrometer (Thermo Scientific) as described elsewhere [56]. The peptide mixtures were injected onto the column in HPLC solvent A (0.1% formic acid) at a flow rate of 500 nl/min and subsequently eluted with an 127 minute segmented gradient of 5–33-50-90% of HPLC solvent B (80% acetonitrile in 0.1% formic acid) at a flow rate of 200 nl/min. The 10 most intense precursor ions were sequentially fragmented in each scan cycle using collision-induced dissociation (CID). In all measurements, sequenced precursor masses were excluded from further selection for 90 s. The target values were 5000 charges for MS/MS fragmentation and 10^6^ charges for the MS scan.

Acquired MS spectra were processed with MaxQuant software package version 1.5.2.8 [57] with integrated Andromeda search engine [58]. Database search was performed against a target-decoy *Arabidopsis thaliana* database obtained from Uniprot, containing 33,431 protein entries and 285 commonly observed contaminants. In database search, full specificity was required for trypsin. Up to two missed cleavages were allowed. Oxidation of methionines and N-terminal acetylation were specified as variable modifications, whereas carbamidomethylation on cysteines was defined as a fixed modification. Initial mass tolerance was set to 4.5 parts per million (ppm) for precursor ions and 0.5 dalton (Da) for fragment ions. Peptide, protein and modification site identifications were reported at a false discovery rate (FDR) of 0.01, estimated by the target/decoy approach [59]. The iBAQ algorithm was enabled to estimate quantitative values by dividing the sum of peptide intensities of all detected peptides by the number of theoretically observable peptides of the matched protein [60].

### *In-vitro* exchange assay

Expression of SEC7^BIG3^ and its ARF substrates, production of recombinant proteins, and fluorescence measurements were performed as described [38]. The catalytic SEC7 domain of BIG5 (residues 587–791) was cloned into pET30a vector (Merck) for expression in *E*. *coli*. For expression of ARFs, DNA fragments corresponding to residues 18–181 of ARF1 isoforms (ARF1-WT, ARF1-T31N) and to residues 18–192 of ARFA (ARFB1b) isoforms (ARFA-WT, ARFA-T31N and ARFA-Q71L) were PCR-amplified from respective templates and cloned into pET30a vector using *NcoI* and *XhoI*. The following primers were used for amplification of ARF1-WT and ARF1-T31N: ARF1d17_pET30_F, CCTTCCATGGGTATGCGTATTCTGATGGTT; ARF1d17_pET30_R, CCTTCTCGAGTTATGCCTTGCTTGCGATGTT. The following primers were used for amplification of ARFA-WT, ARFA-T31N and ARFA-Q71L: ARFAd17_pET30_F, CCTTCCATGGGTATGAGGGTCGTTATGCTG; ARFAd17_pET30_R, CCTTCTCGAGTTAAAACGAGTGGCCAACC.

The recombinant proteins were expressed in BL21 (DE3) strain of *E*. *coli* by inducing with 0.05mM IPTG for 24h at 18°C. The histidine-tagged (6XHis) proteins were purified using HisPur Ni-NTA resin (Thermo Scientific) according to the manufacturer´s instructions.

The *in-vitro* exchange assay was performed as described [61]. Briefly, 25μM histidine tagged ARF protein solution in GDP exchange buffer (20mM HEPES-NaOH (pH7.5), 50mM NaCl, 0.5mM MgCl_2_, 5mM EDTA, 1mM DTT) was added with 24-fold molar excess of GDP (Sigma) and the reaction mixture was incubated for 90min at 20°C. The reaction was stopped by addition of MgCl_2_ to a final concentration of 10mM and a subsequent incubation for 30min at 20°C. Afterwards, the buffer of the protein sample was exchanged to the nucleotide exchange buffer (40mM HEPES-NaOH (pH 7.5), 50mM NaCl, 10mM MgCl_2_, 1mMDTT). For SEC7^BIG5^ mediated exchange measurements, 1μM of ARF proteins in nucleotide exchange buffer and 50nM SEC7^BIG5^ were used. The exchange reaction was initiated by adding 66μM GTPγS at 37°C. The measurement of tryptophan fluorescence was carried out using FS5-Spectrofluorometer (Edinburgh Instruments) at the excitation and emission wavelength of 298nm and 340nm, respectively [38].

### Quantitative transport assays

Protoplasts were prepared and electrotransfected as previously done [47]. Harvesting and analysis of medium and cell samples as well as calculation of the secretion index was performed as described [62].

### Physiological tests

To investigate primary root growth, 5–6 days old seedlings were transferred to plates with 20 μM Estradiol and or 5μM BFA and analyzed after 2 additional days using ImageJ.

For analysis of seed germination, seeds were sown out on 20μM Estradiol or 5μM/7μM BFA containing MS-medium. Images were taken after 5 days of growth.

### Phylogenetic tree

Full-length protein sequence of ARFA1c, ARFB1b and ARFB1a and ARFD was used to search for related sequences from different plant species with sequenced genomes that are available at the NCBI (https://blast.ncbi.nlm.nih.gov/Blast.cgi) or phytozome homepage (http://www.phytozome.net/). ARF sequences from different species were aligned and the phylogenetic tree was drawn with CLC software. Full-length protein sequence of GNOM, GNL1, GNL2, BIG1-BIG5, HsGBF1, HsBIG, HsBIG2 ScGea1, ScGea2 and ScSec7 were aligned and the phylogenetic tree was drawn with CLC.

#### Calculation of Pearson´s correlation coefficient

Pearson´s correlation coefficients of ARF GTPases of classes I, A and B with the TGN-marker VHA-a1-RFP or the Golgi-marker Wave22-mCherry were analyzed using the PSC colocalization ImageJ plug-in (http://www.cpib.ac.uk/~afrench/coloc.html; [63]). The threshold level was set at 0. Because of the high cytosolic amount of ARF GTPases, only ARF-positive endosomes/Golgi were selected for the analysis. For each correlation, 32–69 cells in 3–7 images were analyzed (ARF1-YFP/VHA-a1-RFP: 32 cells in 6 images; ARF1-YFP/ Wave22-mCherry: 52 cells in 7 images; ARFA-YFP/VHA-a1-RFP: 43 cells in 3 images; ARFA-YFP/Wave22-mCherry: 57 cells in 6 images; ARFB-YFP/VHA-a1-RFP: 69 cells in 6 images; ARFB-YFP/Wave22-mCherry: 69 cells in 8 images). The standard deviation between the different images was calculated and included as error bar.

## Supporting information

S1 Fig Phylogenetic analysis of eukaryotic ARF GTPase superfamily and Arabidopsis ARF-GEF family. (**A**) Genome sequences of 16 species representing the five major clades of Eukaryotes ([64]; see S2 Table) were phylogenetically analyzed for the presence of ARF, ARF-like (ARL), ARF-related protein (ARFRP) and SAR1 GTPases. Arabidopsis sequences (asterisks) were used to search the genomes using the BLAST tool of the NCBI (https://blast.ncbi.nlm.nih.gov/Blast.cgi) or Phytozome (https://phytozome.jgi.doe.gov/pz/portal.html) web pages. Obtained sequences were blasted back against Arabidopsis or human genome (see also e-values in S2 Table). Sequence alignment and the phylogenetic tree were generated via CLC software. (**B**) The Arabidopsis ARF-GEF family comprises three GBF1-related ARF-GEFs named GNOM, GNL1 and GNL2, and five ARF-GEFs of the BIG-subfamily named BIG1-BIG5. At, Arabidopsis thaliana; Hs, Homo sapiens; Sc, Saccharomyces cerevisiae. (**C**) Pearson´s and Spearman´s correlation coefficients. ARF1-YFP (ARFA1c), ARFA-YFP (ARFB1b) and ARFB (ARFB1a) and the TGN-marker VHA-a1-RFP or the Golgi-marker Wave22-mCherry were analyzed using the PSC colocalization ImageJ plug-in. For each correlation, 32–69 cells in 3–7 images were analyzed. The error bars indicate the standard deviation between the different images. See also S7 Data.
(PDF)

S2 Fig ARF1 is crucial for embryo development whereas inactivation of ARFA or ARFB does not affect seedling development. (**A-X**) Activation-impaired (T31N; TN) or hydrolysis-impaired (Q71L; QL) variants of ARF1 and ARFA were expressed in the *Gal4>>UAS* two-component system from the *RPS5a* promoter. Embryos were analyzed 5 days (A-C, G-I, M-R, V-X) or 3 days (D-F, J-L, S-U) after pollination of *RPS5a*::*Gal4* driver line with Columbia (Col) wild-type (**A-F**), *UAS*::*ARF1-TN-YFP* (**G-L**), *UAS*::*ARF1-QL-YFP* (**P-U**) reporter lines. Embryos expressing ARF1-TN-YFP or ARF1-QL-YFP showed defects in embryo development with bi- or multinucleate cells. Note that ARF1-TN-YFP causes more severe defects than ARF1-QL-YFP. (**M-O, V-X**) Embryo phenotypes were analyzed after 5 days after pollination of *RPS5a*::*Gal4* driver line with *UAS*::*ARFA-TN-YFP* (**M-O**) or *UAS*::*ARFA-QL-YFP* (**V-X**) reporter lines. ARFA-TN-YFP (**M-O**) did not affect embryo development and was comparable to wild-type (Col) embryos (**A-C**). Expression of ARFA-QL-YFP (**V-X**) caused mild phenotypes of raspberry-shaped embryos, very similar to the expression of ARF1-QL-YFP (**P-R**). Scale bar, 10μm. (**Y**) RT-PCR of T-DNA insertion lines for the two *ARFA* genes, *ARFB1b* (*left*) and *ARFB1c* (*right*). No transcripts of *ARF B1b* or *ARF B1c* were detectable in the respective T-DNA insertion line. C, control. (**Z, A1**) *arfA* double mutant (*B1b B1c;* **Z**) and amiRNA against *ARFB* (*B1a;* **A1**) showed no obvious phenotypes in comparison to Columbia wild-type (WT) control. (**B1**) RT-PCR of 6 independent lines expressing an artificial microRNA (amiRNA) against *ARFB* (*B1a*). No transcript of *ARFB* (*B1a*) was detectable (*left*) whereas expression of *ARFA* (*B1b*) was unaffected (*right*). C, control. (**C1**) Two independent transgenic lines of activation-impaired Est>>ARFA-TN-YFP and Est>>ARFB-TN-YFP transgenic lines were crossed. F2 was germinated on 20μM estradiol. Seedlings harboring both Est>>ARFA-TN-YFP and Est>>ARFB-TN-YFP (asterisks) were identified by PCR-genotyping for the presence of the *T31N* mutation in *ARFA* (*B1b*) and *ARFB* (*B1a*) and did not show any defect in seedling development. Scale bar, 1cm.
(PDF)

S3 Fig Loss of ARFA and ARFB does not affect plant development. One *RPS5A*::*amiR(ARFB)* (*B1a*) expressing line was crossed into *arfA* (*B1b B1c*) double mutant background. (**A**) Flowers from two *arfA* (*B1b B1c*) double mutant plants (deriving from the same cross) that contained the artificial microRNA against *ARFB* (#2–1*, #2–2*), one *arfA* double mutant plant (#1) and Columbia (Col) control were analyzed for *ARFB* (*B1a*) and *ARFA* (*B1b*, *B1c*) transcripts. Col showed transcript for class-A ARFs (*B1b B1c*), class-B ARF (*B1a*) and *Actin*, whereas *arfA* double mutant plant (#1) did not show transcript for the two class-A ARFs (*B1b B1c)*, but showed expression of *ARFB* (*B1a*) and *Actin*. *arfA* double mutant plants harboring the artificial microRNA against *ARFB* showed no transcript for *ARFA (B1b B1c)*, and *ARFB* (*B1a*) but showed expression of *Actin*, representing an *arfA arfB* knockout plant. (**B**) Phenotypes of Columbia, *arfA* double mutant (#1) and two *arfA* double mutant plants expressing *RPS5A*::*amiR(ARFB)* (*arfA arfB* knockout mutant). Scale bar 3cm.
(PDF)

S4 Fig Phenotypic analysis of ARFA-T31N,Q71L double mutant protein expression. (**A-I**) Subcellular localization of ARFA-TN-YFP (A), ARFA-QL-YFP (D) and ARFA-TN,QL-YFP (G) expressed from the Estradiol (Est)-inducible system and the endocytic tracer FM4-64 after 6h Estradiol treatment (B, E, H). ARFA-TN-YFP partially colocalizes with FM4-64 in a punctate pattern (A-C), whereas ARFA-QL-YFP colocalizes with FM4-64 in aggregates (D-F). ARFA-TN,QL-YFP shows a punctuate pattern that partially overlaps with FM4-64 (G-I) similar to ARFA-TN-YFP lines. Scale bar 5μM. (**J**) Effect of ARFA-TN-YFP, ARFA-QL-YFP and ARFA-TN,QL-YFP expression on primary root growth. 5 days old seedlings were transferred onto plates with (+) and without (-) 20μM Estradiol and primary roots were measured after 2 days. Error bars represent standard deviation. Number of seedlings analyzed is indicated above respective error bars. (K) Expression of Estradiol induced ARFA-TN-YFP, ARFA-QL-YFP and ARFA-TN,QL-YFP was analyzed by Western blot using αGFP antibody (upper part, asterisk). Ponceau S staining was used as loading control (lower panel). (**L**) Estradiol inducible ARFA-TN-YFP in *big3* mutant background was crossed into UBQ10 driven BFA-resistant BIG4-YFP in *big3* mutant background (UBQ10::BIG4^R^-YFP *big3*). 5 days old F1 seedlings were transferred to 5μM BFA or 5μM BFA plus 20μM Estradiol containing plates and were analysed for primary root growth after 2 days. Primary root growth on BFA and BFA plus EST was compared. Error bars display standard deviation. Number of seedlings analyzed are depicted above the respective error bars. See also S8 and S9 Data.
(PDF)

S5 Fig ARF1 but not ARFA is essential for secretion, ER morphology and endocytosis. Expression of activation-impaired YFP-tagged (T31N; TN; green) variants of ARF1 and ARFA was induced with 20μM estradiol for 5-6h and trafficking markers (magenta) were analyzed in live-cell imaging (A-I; R-E1) or immunostaining (J-Q). (**A, B**) N-ST-YFP localized at the Golgi in wild-type (Col; A) but re-localized to the ER in an untagged ARF1-TN (B) expressing line. (**C-I**) SYP132 expressed from the *Histone 4* promoter (H4::RFP-SYP132) localized at the plasma membrane in wild-type (Col; C), ARF1-TN-YFP (D-F) and ARFA-TN-YFP (G-I). In contrast, intracellular accumulation of RFP-SYP132 was only observed in ARF1-TN-YFP lines (D-F). (**J-Q**) The cytokinesis-specific syntaxin KNOLLE localized at the cell plate in ARFA-TN-YFP (N-Q) expressing lines. Tubulin staining visualized phragmoplasts during cytokinesis (L, P; cyan). KNOLLE (J) was not transported to the cell plate in ARF1-TN-YFP but localized in the ER (J-M). blue, DAPI stained nuclei. (**R-X**) The ER was marked by sec-HDEL-mCherry (R, S, V). In ARF A-TN-YFP (V-X), ER morphology was comparable to the wild-type control (Col; R). In contrast, ARF1-TN-YFP (S-U) severely altered the ER morphology to a ball shape. (**Y-E1**) BFA/ estradiol-treated (50μM BFA; 20μM estradiol) seedling root cells in which RFP-PEN1 (Y; Z; C1) expressed from the *Histone 4* promoter localized in BFA-compartments in wild-type (Y), ARFA-TN-YFP (C1-E1) and ARF1-TN-YFP (Z-B1). These lines were used for BFA wash-out experiments shown in Figs 3 and 4. Scale bar 10μm. The same wild-type controls were used as in Figs 3 and 5 and S5.
(PDF)

S6 Fig Secretion of xyloglucan to the extracellular space is affected by interference with ARF1 function. Xyloglucan immuno-labelling in root cells, using ultrathin resin sections from high-pressure frozen, freeze-substituted and Lowicryl-embedded seedling root tips. Xyloglucan serves as a secretory marker. Ultrathin resin sections were double labelled with fluorescent (Cy3) and gold (6 nm gold) markers for correlative light and electron microscopy (CLEM). (**A-L**) A single section is double labelled. (**M-X**) Consecutive sections from the identical root tip were labelled either with Cy3 or with gold marker. (**A-L**) ARF1-TN-YFP. (**A**) Root tip region (overview). (**B, C**) Enlarged boxed area of (A) as overlay with TEM image, showing a large number of homogeneously sized fluorescent vesicles in a root cap cell. (**D**) Enlarged area of (B, C) as TEM image with gold-labelled vesicles. (**E**) Enlarged area of (D) showing gold marker (circles). (**F**) Root region, contiguous part of (A) (same section), showing larger xyloglucan-positive structures. (**G, H**) Enlarged boxed area of (F) as overlay with TEM image. (**I**) Enlarged boxed area of (G, H) as overlay with TEM image (cortex cell). (**K**) Enlarged boxed area of (I) as TEM image with gold-labelled ER-connected compartment. (**L**) Enlarged boxed area of (K) showing gold marker (circles). (**M-R**) ARF1-QL-YFP. (**M, N**) Root tip region, comparable to (F) (overview). (**M**) overlay with DNA staining (blue), (**N**) without DNA staining; strong clustering of fluorescent signal can be detected. (**O, P**) Two enlarged cells (boxed area in (N)), (**P**) shows additional weak vacuolar staining (circles). (**Q**) Representative vesicle cluster showing strong gold labelling, enlarged inset showing single vesicle. (**R**) Golgi stack located in vesicle cluster with gold labelled vesicles; enlarged inset showing single gold-labeled vesicle. (**S-U**) ARFA-TN-YFP. (**S, T**) Root tip region, comparable to (F) (overview). (**T**) Enlarged boxed area of (S) showing weak clustering of fluorescent spots. (**U**) TEM image of representative region with clustered gold signal, located at the TGN; enlarged inset showing two gold-labelled vesicles. (**V-X**) ARFA-QL-YFP. (**V, W**) Root tip region, comparable to (F) (overview). (**W**) Enlarged boxed area of (S) showing stronger clustering of fluorescent spots. (**X**) TEM image of representative region with gold signal in clustered vesicles; enlarged inset showing gold-labelled vesicle. Abbreviations: c, cortex; cw, cell wall; e, epidermis; er, endoplasmic reticulum; erc, ER-connected compartment; g, Golgi stack; n, nucleus; rc, root cap; t, trans-Golgi network; v, vesicle; va, vacuole. Scale bars represent 500 nm in (D, K, Q, R, U, X); 10 μm in (A, B, C, F, G, H, M, N, S, V).
(PDF)

S7 Fig Expression of ARF1-Q71L and ARFA-Q71L interferes with secretion but not with ER morphology and endocytosis. Expression of hydrolysis-impaired, YFP-tagged (Q71L; QL; green) variants of ARF1 and ARFA was induced by 20μM estradiol for 5-6h and trafficking markers (magenta) were analyzed in live-cell imaging (A-G; P-C1) or immunostaining (H-O). (**A-G**) The ER was marked by sec-mCherry-HDEL (A, B, E). In ARF1-QL-YFP (B-D) and ARFA-QL-YFP (E-G) ER morphology was comparable to the wild-type control (Col; A). (**H-O**) The cytokinesis-specific syntaxin KNOLLE localized at the cell plate (asterisk) in some cells of ARFA-QL-YFP (L-O) expressing lines. Tubulin staining visualized phragmoplasts during cytokinesis (J, N; cyan). KNOLLE was not transported to the cell plate but localized in intracellular patches in some cells of ARF1-QL-YFP (H-K, arrowhead) and not in ARFA-QL-YFP lines (L-O; arrowhead). Blue, DAPI-stained nuclei. (**P-V**) SYP132 expressed from the *Histone 4* promoter (H4::RFP-SYP132) localized at the plasma membrane in wild-type (Col; P), ARF1-QL-YFP (Q-S) and ARFA-QL-YFP (T-V). In addition, intracellular accumulation of RFP-SYP132 was observed in ARF1-TN-YFP (Q-S) and ARFA-QL-YFP (T-V) lines but not in wild-type (P). (**W-C1**) BFA treatment (1h 50μM) was used to visualize endocytosed RFP-PEN1 (Y, X, A1) in BFA compartments. Endocytosis of PEN1 was comparable to wild-type control (W) in ARF 1-QL-YFP (X-Z) and ARFA-QL-YFP (A1-C1). Scale bar, 10μm. The same wild-type controls were used as in Figs 3 and 5 and S3.
(PDF)

S8 Fig Interaction of ARF1, ARFA and ARFB with different ARF-GEFs and localization of ARFA-YFP in *big5* mutant. (**A**) The α-ARF1 antiserum did not recognize ARFA and ARFB. Total protein from wild-type (Col-0) and the transgenic plants expressing ARF1-YFP, ARFA-YFP and ARFB-YFP were separated on SDS-PAGE followed by western blot using α-ARF1 (IB: α-ARF1) or α-GFP (IB:α-GFP) antibody. (**B**) GNOM-ARF1 interaction was enhanced in the presence of Brefeldin A (BFA). Immunoprecipitation (IP) was performed using GFP-Trap-agarose beads (IP: α-GFP) from transgenic plants co-expressing ARF1-YFP and GNOM-Myc (GN-Myc), in the presence or absence of BFA. IP was followed by immunoblot (IB) analysis using α-Myc antibody. (**C**) Co-immunoprecipitation of ARF1 with GNL2. Immunoprecipitation (IP) of GNL2 using GFP-Trap-agarose beads (IP: α-GFP) from transgenic plants expressing GNL2-GFP under the control of the *GNOM* promoter (*GN*::*GNL2-GFP*), was followed by immunoblot (IB) analysis using α-ARF1 antibody. Col-0, wild-type control. (**D**) Co-immunoprecipitation of ARF1 with BIG5. Immunoprecipitation (IP) of BIG5 using GFP-Trap-agarose beads (IP: α-GFP) from transgenic plants expressing BIG5-YFP was followed by immunoblot (IB) analysis using α-ARF1 antibody. Col-0, wild-type control. (**E**) ARFA (ARFB1b)-YFP did not co-immunoprecipitate with GNOM. Immunoprecipitation of GNOM-Myc (GN-Myc) from transgenic plants co-expressing ARFA-YFP and GNOM-Myc using α-Myc-agarose beads was followed by immunoblot (IB) analysis using α-GFP antibody and α-ARF1 antibody to detect YFP-tagged ARFA and endogenous ARF1, respectively. The protein band near 50 kDa in the IP lane corresponds to Ig heavy chain from α-Myc-agarose. (**F**) ARFA (ARFB1c)-RFP did not co-immunoprecipitate with GNOM. Immunoprecipitation(IP) of GNOM-Myc (GN-Myc) from transgenic plants co-expressing ARFA-RFP and GNOM-Myc was performed with α-Myc-agarose beads followed by immunoblot (IB) analysis using α-RFP antibody and α-ARF1 antibody. (**G**) ARFA (ARFB1b)-YFP did not co-immunoprecipitate with GNL1. Immunoprecipitation of GNL1-Myc from transgenic plants co-expressing ARFA-YFP and GNL1-Myc using α-Myc-agarose beads was followed by immunoblot (IB) analysis using α-GFP antibody and α-ARF1 antibody. (**H**) ARFA (ARFB1c)-RFP did not co-immunoprecipitate with BIG3. Immunoprecipitation (IP) of BIG3 using GFP-Trap-agarose beads (IP: α-GFP) from transgenic plants co-expressing BIG3-YFP and ARFA-RFP was followed by immunoblot (IB) analysis using α-RFP antibody and α-ARF1 antibody. (**I**) ARF1-T31N (TN)-YFP but not ARF1-Q71L (QL)-YFP co-immunoprecipitated with GNOM. Immunoprecipitation (IP) of ARF1-TN-YFP and ARF1-QL-YFP using GFP-Trap-agarose beads (IP: α-GFP) from transgenic plants expressing ARF1-TN-YFP or ARF1-QL-YFP was followed by immunoblot (IB) analysis using α-SEC7(GNOM) antibody. (**J**) ARFA-T31N (TN)-YFP but not ARFA-Q71L (QL)-YFP co-immunoprecipitated with BIG5^R^. Immunoprecipitation (IP) of BIG5^R^-RFP (BFA-resistant version of BIG5) using RFP-Trap-agarose beads (IP: α-RFP) from transgenic plants co-expressing BIG5^R^-RFP either with ARFA-TN-YFP or with ARFA-QL-YFP was followed by immunoblot (IB) analysis using α-GFP antibody and α-ARF1 antibody. IN, input; IP, immunoprecipitate; IB, immunoblot; kDa, kilodalton. (**K-N**) ARFA-YFP localization in wild-type (WT) (**K-L**) and *big5* mutant (**M-N**) after BFA treatment. Seedlings were treated with BFA (50μM) for 1h. Scale bars, 10μm.
(PDF)

S9 Fig Expression of recombinant proteins and *in vitro* exchange assay. (**A-B**) Effect of catalytic SEC7 domain of BIG5 (SEC7^BIG5^) on nucleotide exchange rate of ARF1 (ARFA1c) isoforms (A) and ARFA (ARFB1b) isoforms (B). The increase in tryptophan fluorescence over time reflects a conformational change from GDP-bound state to GTPγS-bound state. The blue line represents exchange activity of GEF (SEC7^BIG5^) on ARF1 wild-type (ARF1-WT) (A) and ARFA wild-type (ARFA-WT) (B) in absence of GTPγS whereas the red line represents spontaneous exchange activity of ARF1-WT (A) and ARFA-WT (B) in absence of GEF (SEC7^BIG5^). The green line shows GDP-GTP exchange activity of SEC7^BIG5^ on ARF1-WT (A) and ARFA-WT (B) whereas the purple line represents exchange activity of SEC7^BIG5^ on ARF1-T31N (A) and ARFA-T31N (B). The orange line shows GDP-GTP exchange activity of SEC7^BIG5^ on ARFA-Q71L protein (B). The experiments were performed using 1μM ARF, 50nM SEC7^BIG5^ and 66μM GTPγS at 37°C. The tryptophan fluorescence was measured at an interval of 5 seconds, using the excitation and emission wavelength of 298nm and 340nm, respectively. Note that both ARF1-T31N and ARFA-T31N do not show any increase in tryptophan fluorescence during the course of measurement. (**C-D**) Coomassie-stained SDS-PAGE showing expression and purification of His (6X)-tagged ARF1-WT, ARF1-T31N (TN), SEC7^BIG5^ (C) and ARFA-WT, ARFA-T31N (TN), ARFA-Q71L (QL) (D) using Ni-NTA resin. T, total; S, soluble fraction; P, purified protein (from Ni-NTA resin); kDa, kilodalton. See also S10 Data.
(PDF)

S1 Table Evolutionary distribution of ARFs and ARLs within the Archaeplastida: Isoform numbers and sequence identity to Arabidopsis proteins. (DOCX)

S2 Table Eukaryotic ARF GTPase family proteins, accession numbers and relatedness to Arabidopsis and human homologs. (XLSX)

S3 Table Mass spectrometric analysis of ARF-interacting ARF-GEFs. Sheet 1 contains the relevant candidates taken from the complete list of identification in sheet 2. Sheet 2 contains identified protein groups sorted by gene names and presented together with their iBAQ (intensity based absolute quantification: sum of peak intensities of all peptides matching to a specific protein divided by the number of theoretically observable peptides) values. See also PRIDE repository, dataset identifier PXD011594 (https://www.ebi.ac.uk/pride/archive/)
(XLSX)

S1 Data Numerical data related to Fig 2A. (XLSX)

S2 Data Numerical data related to Fig 2Q. (XLSX)

S3 Data Numerical data related to Fig 2R. (XLSX)

S4 Data Numerical data related to Fig 2S. (XLSX)

S5 Data Numerical data related to Fig 6K. (XLSX)

S6 Data Numerical data related to Fig 7. (XLSX)

S7 Data Numerical data related to S1C Fig. (XLSX)

S8 Data Numerical data related to S4J Fig. (XLSX)

S9 Data Numerical data related to S4L Fig. (XLSX)

S10 Data Numerical data related to S9 Fig. (XLSX)
