# Supplementary figures and images for: A single class of ARF GTPase activated by several pathway-specific ARF-GEFs regulates essential membrane traffic in Arabidopsis
Source: PLoS Genet. 2018 Nov 15;14(11):e1007795. doi: 10.1371/journal.pgen.1007795 (PMC6264874; doi:10.1371/journal.pgen.1007795)

A

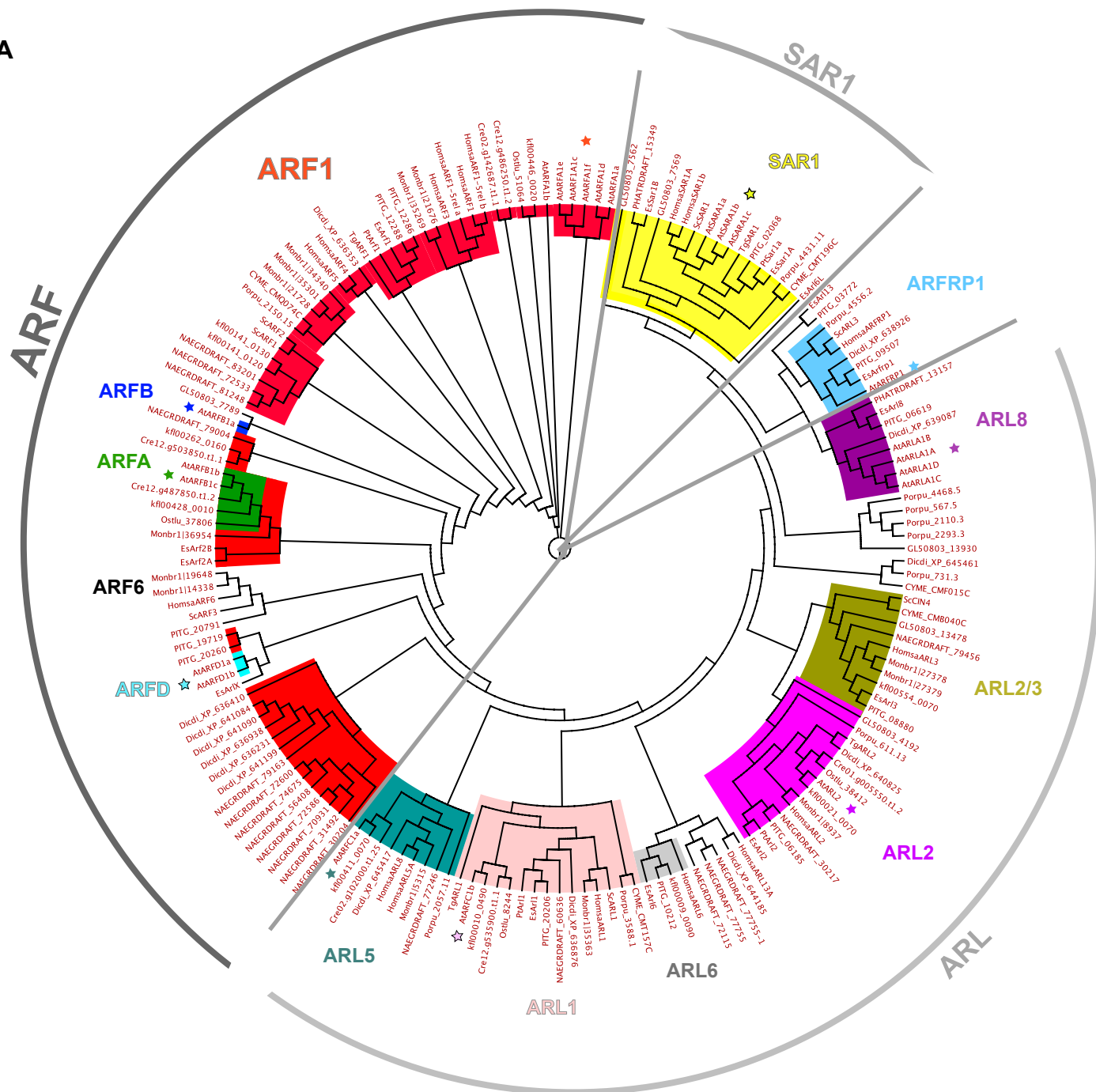

B

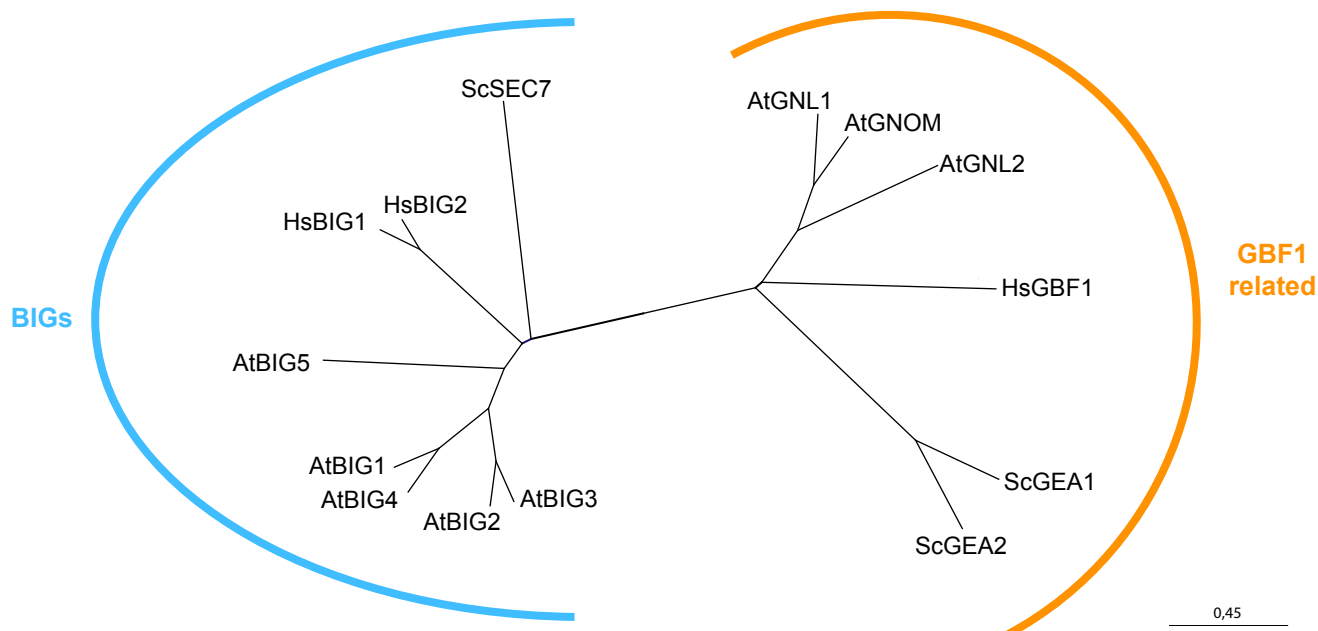

0,45

Supplementary Figure S1

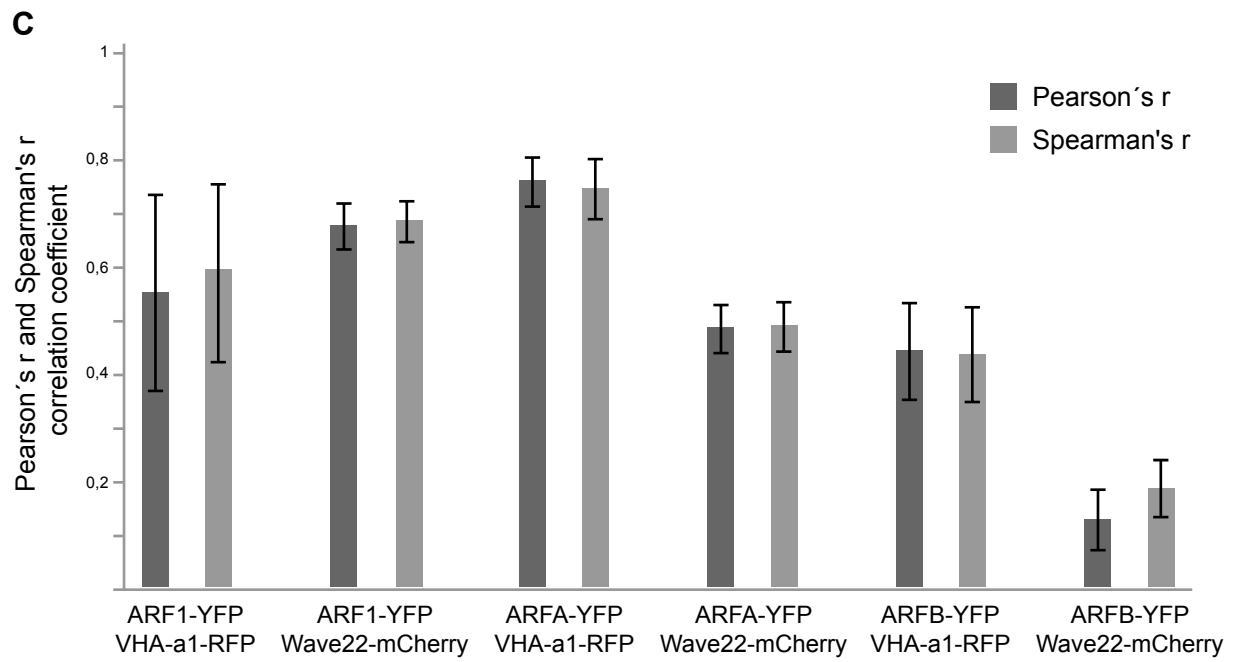

**Supplementary Figure S1**

Supplement: S1 Fig — (A) Genome sequences of 16 species representing the five major clades of Eukaryotes ([64]; see S2 Table) were phylogenetically analyzed for the presence of ARF, ARF-like (ARL), ARF-related protein (ARFRP) and SAR1 GTPases. Arabidopsis sequences (asterisks) were used to search the genomes using the BLAST tool of the NCBI (https://blast.ncbi.nlm.nih.gov/Blast.cgi) or Phytozome (https://phytozome.jgi.doe.gov/pz/portal.html) web pages. Obtained sequences were blasted back against Arabidopsis or human genome (see also e-values in S2 Table). Sequence alignment and the phylogenetic tree were generated via CLC software. (B) The Arabidopsis ARF-GEF family comprises three GBF1-related ARF-GEFs named GNOM, GNL1 and GNL2, and five ARF-GEFs of the BIG-subfamily named BIG1-BIG5. At, Arabidopsis thaliana; Hs, Homo sapiens; Sc, Saccharomyces cerevisiae. (C) Pearson´s and Spearman´s correlation coefficients. ARF1-YFP (ARFA1c), ARFA-YFP (ARFB1b) and ARFB (ARFB1a) and the TGN-marker VHA-a1-RFP or the Golgi-marker Wave22-mCherry were analyzed using the PSC colocalization ImageJ plug-in. For each correlation, 32–69 cells in 3–7 images were analyzed. The error bars indicate the standard deviation between the different images. See also S7 Data. (PDF) [file pgen.1007795.s001.pdf]

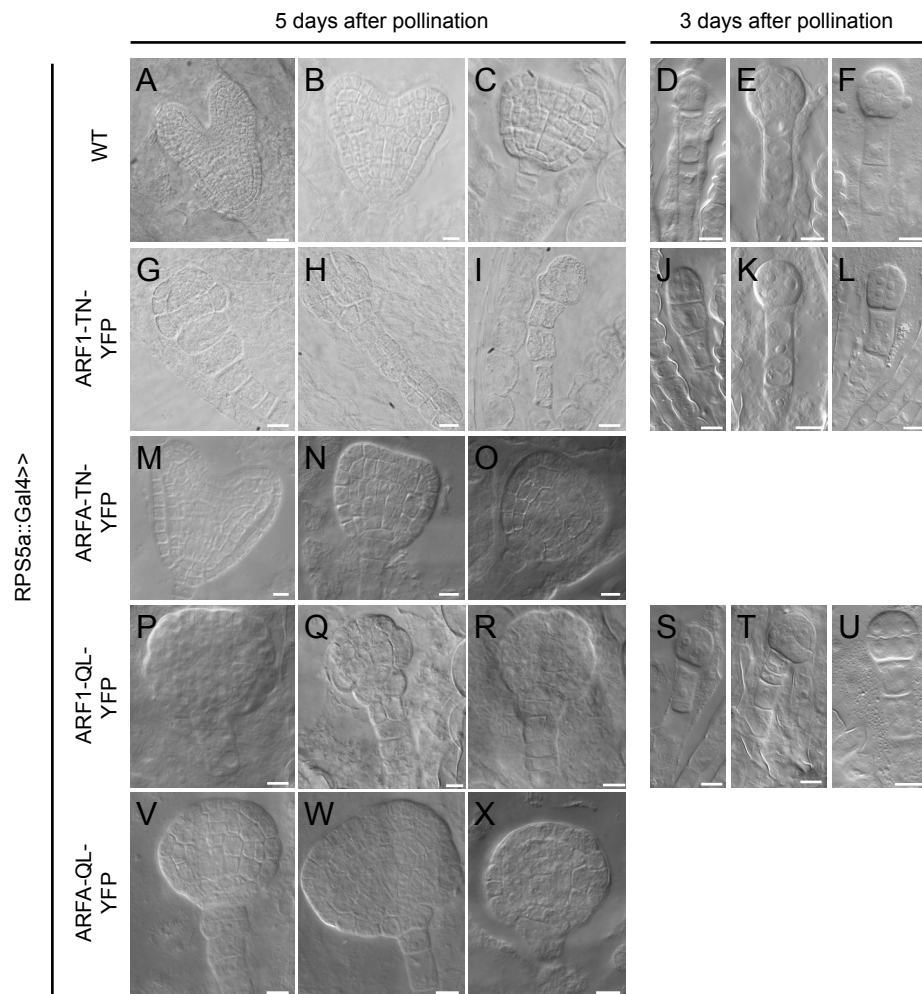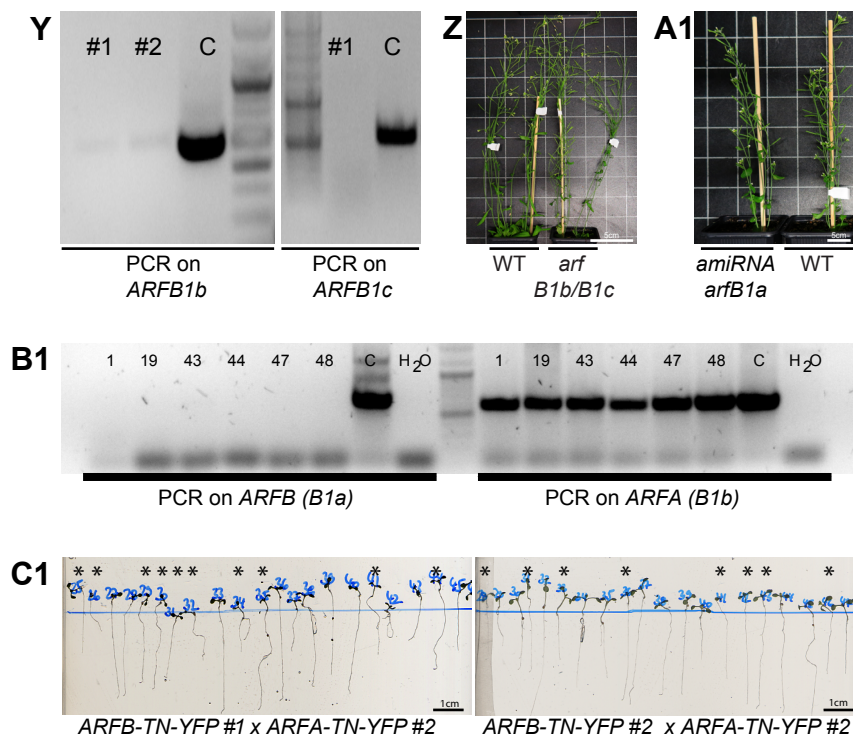

**Supplementary Figure S2**

Supplement: S2 Fig — (A-X) Activation-impaired (T31N; TN) or hydrolysis-impaired (Q71L; QL) variants of ARF1 and ARFA were expressed in the Gal4>>UAS two-component system from the RPS5a promoter. Embryos were analyzed 5 days (A-C, G-I, M-R, V-X) or 3 days (D-F, J-L, S-U) after pollination of RPS5a::Gal4 driver line with Columbia (Col) wild-type (A-F), UAS::ARF1-TN-YFP (G-L), UAS::ARF1-QL-YFP (P-U) reporter lines. Embryos expressing ARF1-TN-YFP or ARF1-QL-YFP showed defects in embryo development with bi- or multinucleate cells. Note that ARF1-TN-YFP causes more severe defects than ARF1-QL-YFP. (M-O, V-X) Embryo phenotypes were analyzed after 5 days after pollination of RPS5a::Gal4 driver line with UAS::ARFA-TN-YFP (M-O) or UAS::ARFA-QL-YFP (V-X) reporter lines. ARFA-TN-YFP (M-O) did not affect embryo development and was comparable to wild-type (Col) embryos (A-C). Expression of ARFA-QL-YFP (V-X) caused mild phenotypes of raspberry-shaped embryos, very similar to the expression of ARF1-QL-YFP (P-R). Scale bar, 10μm. (Y) RT-PCR of T-DNA insertion lines for the two ARFA genes, ARFB1b (left) and ARFB1c (right). No transcripts of ARF B1b or ARF B1c were detectable in the respective T-DNA insertion line. C, control. (Z, A1) arfA double mutant (B1b B1c; Z) and amiRNA against ARFB (B1a; A1) showed no obvious phenotypes in comparison to Columbia wild-type (WT) control. (B1) RT-PCR of 6 independent lines expressing an artificial microRNA (amiRNA) against ARFB (B1a). No transcript of ARFB (B1a) was detectable (left) whereas expression of ARFA (B1b) was unaffected (right). C, control. (C1) Two independent transgenic lines of activation-impaired Est>>ARFA-TN-YFP and Est>>ARFB-TN-YFP transgenic lines were crossed. F2 was germinated on 20μM estradiol. Seedlings harboring both Est>>ARFA-TN-YFP and Est>>ARFB-TN-YFP (asterisks) were identified by PCR-genotyping for the presence of the T31N mutation in ARFA (B1b) and ARFB (B1a) and did not show any defect in seedling development. Scale bar, 1cm. (PDF) [file pgen.1007795.s002.pdf]

**A**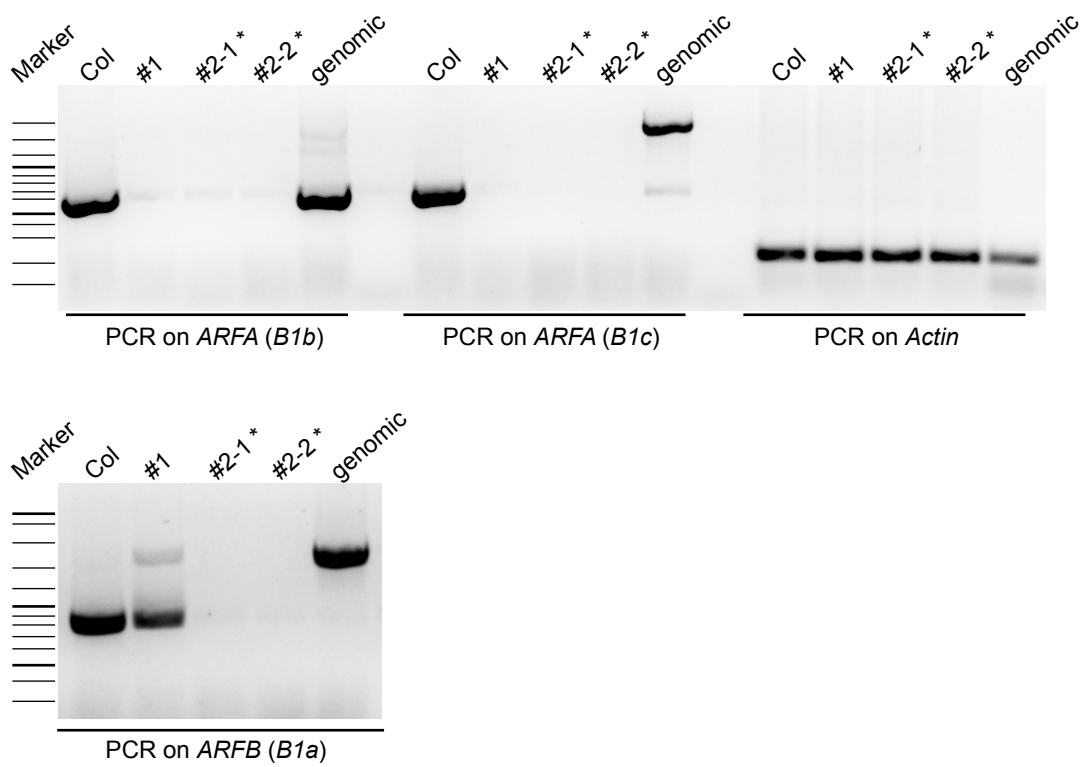**B**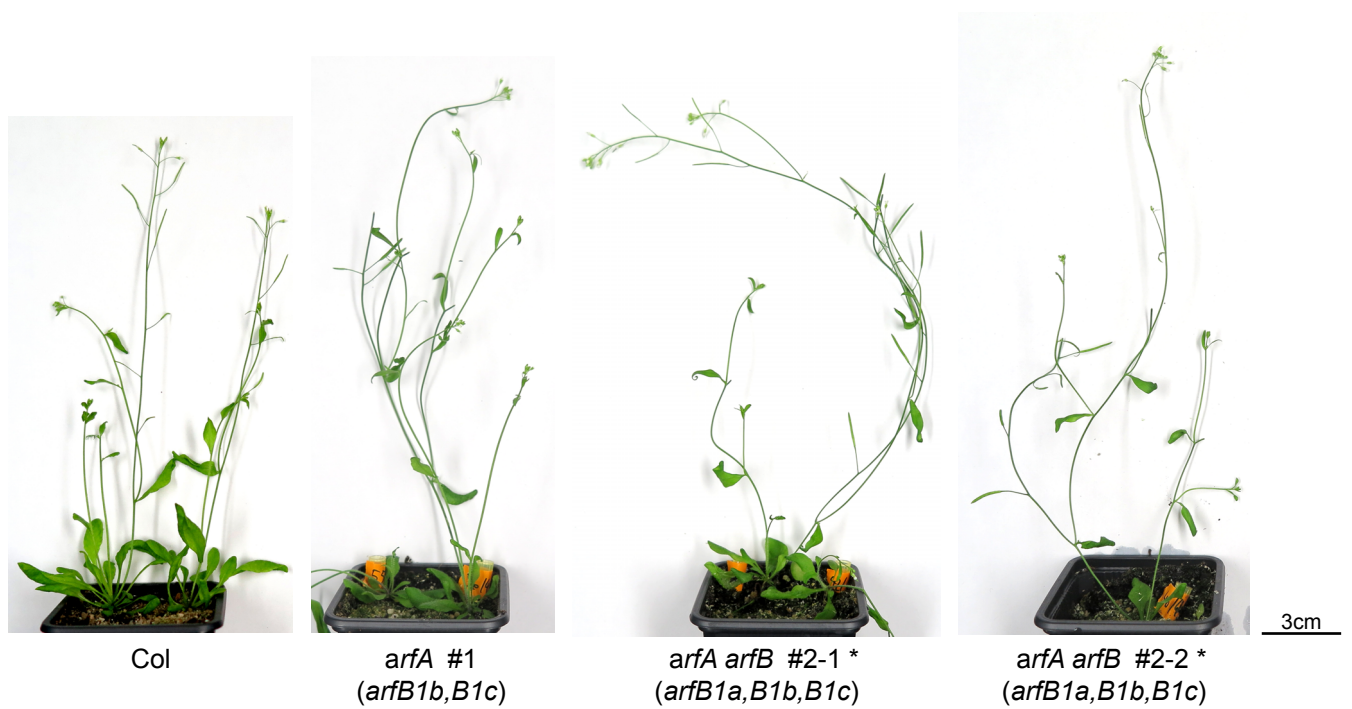**Supplementary Figure S3**

Supplement: S3 Fig — One RPS5A::amiR(ARFB) (B1a) expressing line was crossed into arfA (B1b B1c) double mutant background. (A) Flowers from two arfA (B1b B1c) double mutant plants (deriving from the same cross) that contained the artificial microRNA against ARFB (#2–1*, #2–2*), one arfA double mutant plant (#1) and Columbia (Col) control were analyzed for ARFB (B1a) and ARFA (B1b, B1c) transcripts. Col showed transcript for class-A ARFs (B1b B1c), class-B ARF (B1a) and Actin, whereas arfA double mutant plant (#1) did not show transcript for the two class-A ARFs (B1b B1c), but showed expression of ARFB (B1a) and Actin. arfA double mutant plants harboring the artificial microRNA against ARFB showed no transcript for ARFA (B1b B1c), and ARFB (B1a) but showed expression of Actin, representing an arfA arfB knockout plant. (B) Phenotypes of Columbia, arfA double mutant (#1) and two arfA double mutant plants expressing RPS5A::amiR(ARFB) (arfA arfB knockout mutant). Scale bar 3cm. (PDF) [file pgen.1007795.s003.pdf]

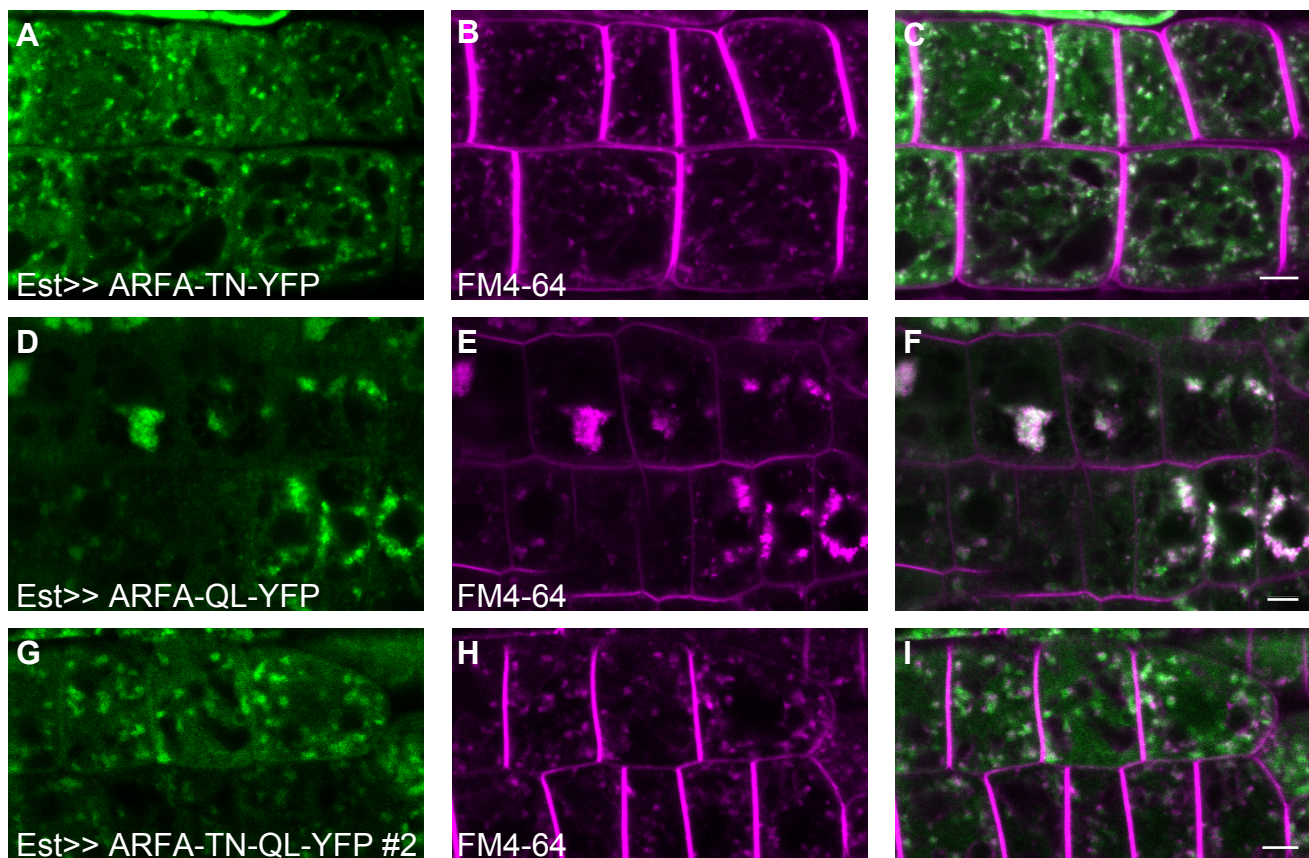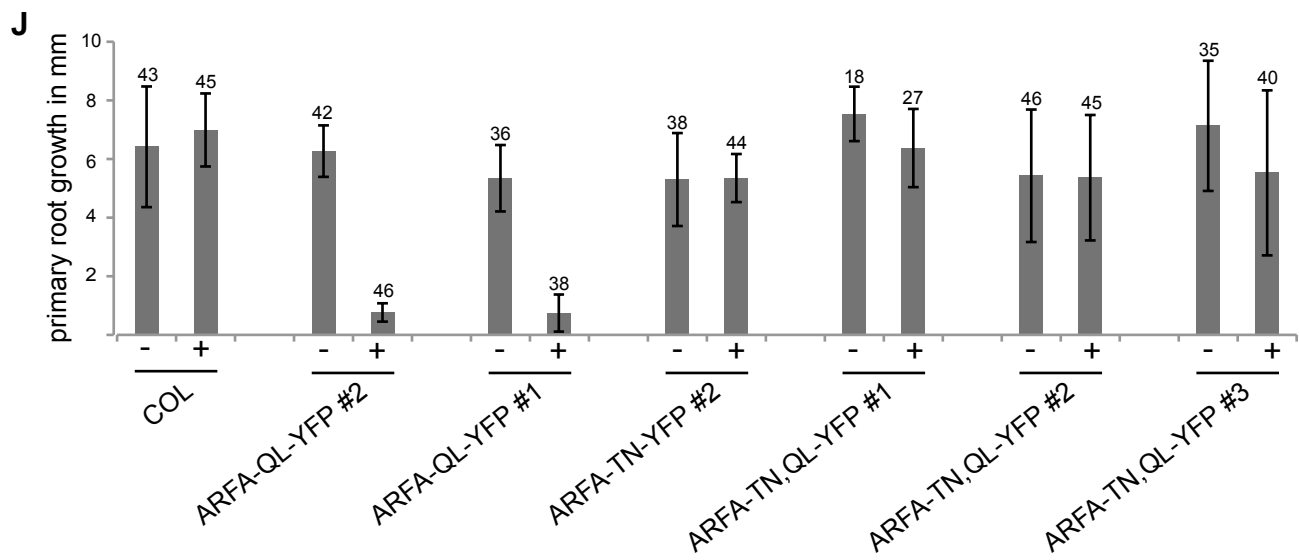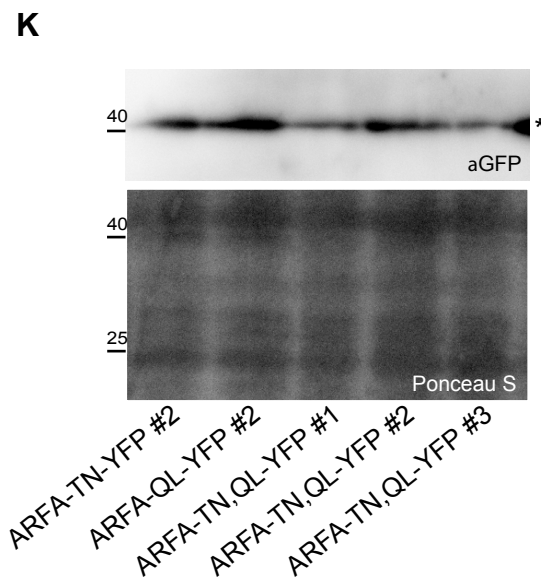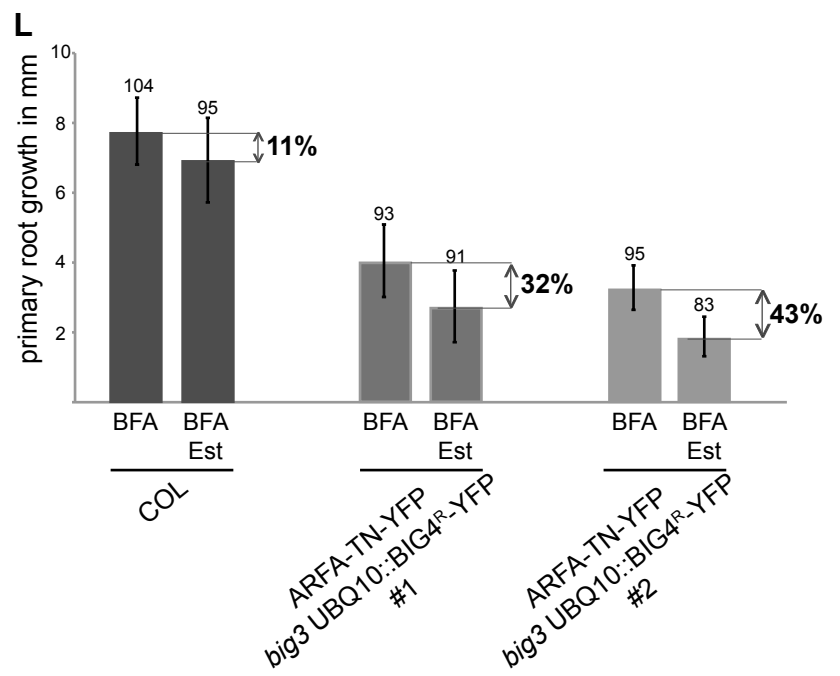

Supplementary Figure S4

Supplement: S4 Fig — (A-I) Subcellular localization of ARFA-TN-YFP (A), ARFA-QL-YFP (D) and ARFA-TN,QL-YFP (G) expressed from the Estradiol (Est)-inducible system and the endocytic tracer FM4-64 after 6h Estradiol treatment (B, E, H). ARFA-TN-YFP partially colocalizes with FM4-64 in a punctate pattern (A-C), whereas ARFA-QL-YFP colocalizes with FM4-64 in aggregates (D-F). ARFA-TN,QL-YFP shows a punctuate pattern that partially overlaps with FM4-64 (G-I) similar to ARFA-TN-YFP lines. Scale bar 5μM. (J) Effect of ARFA-TN-YFP, ARFA-QL-YFP and ARFA-TN,QL-YFP expression on primary root growth. 5 days old seedlings were transferred onto plates with (+) and without (-) 20μM Estradiol and primary roots were measured after 2 days. Error bars represent standard deviation. Number of seedlings analyzed is indicated above respective error bars. (K) Expression of Estradiol induced ARFA-TN-YFP, ARFA-QL-YFP and ARFA-TN,QL-YFP was analyzed by Western blot using αGFP antibody (upper part, asterisk). Ponceau S staining was used as loading control (lower panel). (L) Estradiol inducible ARFA-TN-YFP in big3 mutant background was crossed into UBQ10 driven BFA-resistant BIG4-YFP in big3 mutant background (UBQ10::BIG4R-YFP big3). 5 days old F1 seedlings were transferred to 5μM BFA or 5μM BFA plus 20μM Estradiol containing plates and were analysed for primary root growth after 2 days. Primary root growth on BFA and BFA plus EST was compared. Error bars display standard deviation. Number of seedlings analyzed are depicted above the respective error bars. See also S8 and S9 Data. (PDF) [file pgen.1007795.s004.pdf]

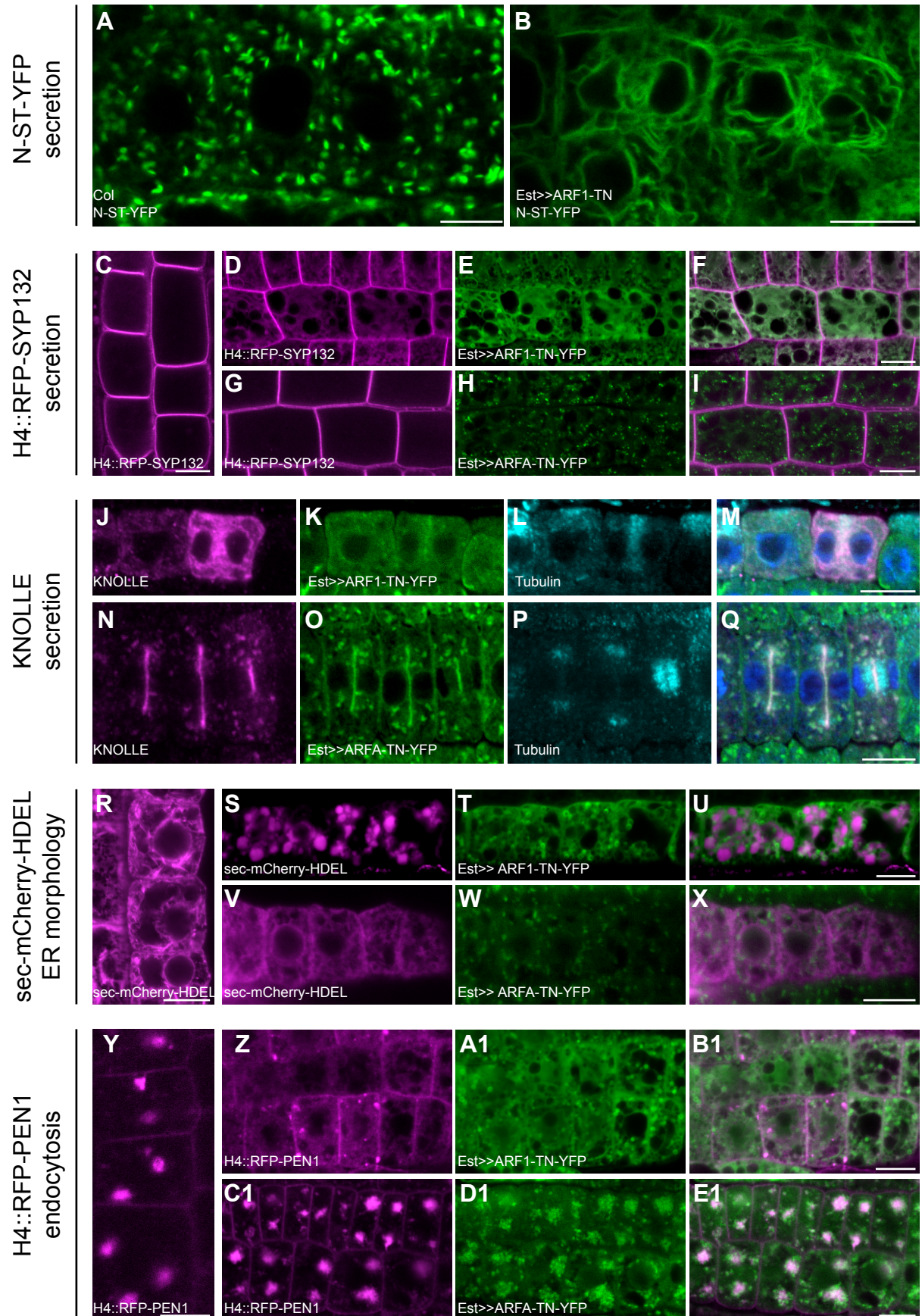

Supplementary Figure S5

Supplement: S5 Fig — Expression of activation-impaired YFP-tagged (T31N; TN; green) variants of ARF1 and ARFA was induced with 20μM estradiol for 5-6h and trafficking markers (magenta) were analyzed in live-cell imaging (A-I; R-E1) or immunostaining (J-Q). (A, B) N-ST-YFP localized at the Golgi in wild-type (Col; A) but re-localized to the ER in an untagged ARF1-TN (B) expressing line. (C-I) SYP132 expressed from the Histone 4 promoter (H4::RFP-SYP132) localized at the plasma membrane in wild-type (Col; C), ARF1-TN-YFP (D-F) and ARFA-TN-YFP (G-I). In contrast, intracellular accumulation of RFP-SYP132 was only observed in ARF1-TN-YFP lines (D-F). (J-Q) The cytokinesis-specific syntaxin KNOLLE localized at the cell plate in ARFA-TN-YFP (N-Q) expressing lines. Tubulin staining visualized phragmoplasts during cytokinesis (L, P; cyan). KNOLLE (J) was not transported to the cell plate in ARF1-TN-YFP but localized in the ER (J-M). blue, DAPI stained nuclei. (R-X) The ER was marked by sec-HDEL-mCherry (R, S, V). In ARF A-TN-YFP (V-X), ER morphology was comparable to the wild-type control (Col; R). In contrast, ARF1-TN-YFP (S-U) severely altered the ER morphology to a ball shape. (Y-E1) BFA/ estradiol-treated (50μM BFA; 20μM estradiol) seedling root cells in which RFP-PEN1 (Y; Z; C1) expressed from the Histone 4 promoter localized in BFA-compartments in wild-type (Y), ARFA-TN-YFP (C1-E1) and ARF1-TN-YFP (Z-B1). These lines were used for BFA wash-out experiments shown in Figs 3 and 4. Scale bar 10μm. The same wild-type controls were used as in Figs 3 and 5 and S5. (PDF) [file pgen.1007795.s005.pdf]

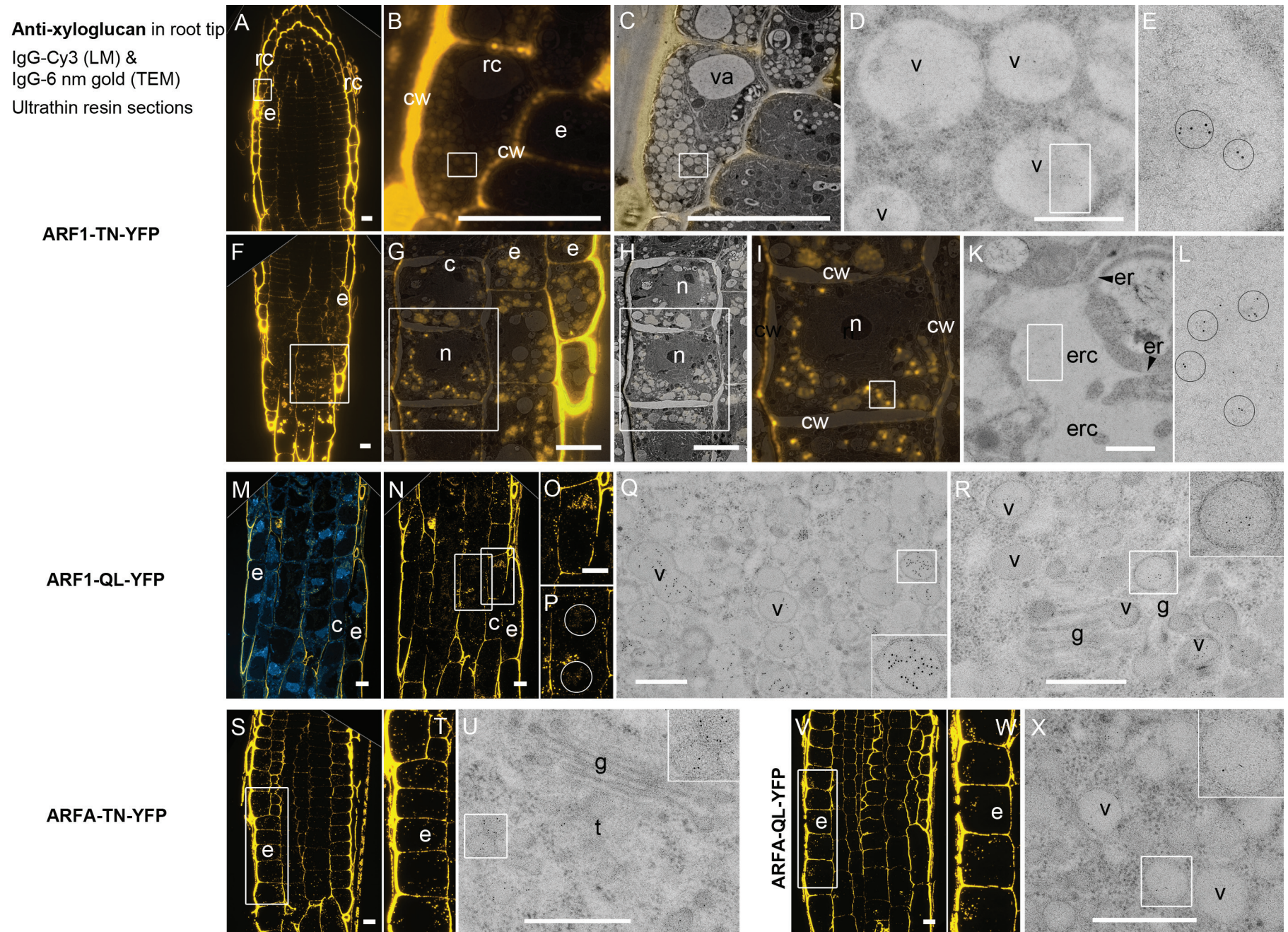

**Supplementary Figure S6**

Supplement: S6 Fig — Xyloglucan immuno-labelling in root cells, using ultrathin resin sections from high-pressure frozen, freeze-substituted and Lowicryl-embedded seedling root tips. Xyloglucan serves as a secretory marker. Ultrathin resin sections were double labelled with fluorescent (Cy3) and gold (6 nm gold) markers for correlative light and electron microscopy (CLEM). (A-L) A single section is double labelled. (M-X) Consecutive sections from the identical root tip were labelled either with Cy3 or with gold marker. (A-L) ARF1-TN-YFP. (A) Root tip region (overview). (B, C) Enlarged boxed area of (A) as overlay with TEM image, showing a large number of homogeneously sized fluorescent vesicles in a root cap cell. (D) Enlarged area of (B, C) as TEM image with gold-labelled vesicles. (E) Enlarged area of (D) showing gold marker (circles). (F) Root region, contiguous part of (A) (same section), showing larger xyloglucan-positive structures. (G, H) Enlarged boxed area of (F) as overlay with TEM image. (I) Enlarged boxed area of (G, H) as overlay with TEM image (cortex cell). (K) Enlarged boxed area of (I) as TEM image with gold-labelled ER-connected compartment. (L) Enlarged boxed area of (K) showing gold marker (circles). (M-R) ARF1-QL-YFP. (M, N) Root tip region, comparable to (F) (overview). (M) overlay with DNA staining (blue), (N) without DNA staining; strong clustering of fluorescent signal can be detected. (O, P) Two enlarged cells (boxed area in (N)), (P) shows additional weak vacuolar staining (circles). (Q) Representative vesicle cluster showing strong gold labelling, enlarged inset showing single vesicle. (R) Golgi stack located in vesicle cluster with gold labelled vesicles; enlarged inset showing single gold-labeled vesicle. (S-U) ARFA-TN-YFP. (S, T) Root tip region, comparable to (F) (overview). (T) Enlarged boxed area of (S) showing weak clustering of fluorescent spots. (U) TEM image of representative region with clustered gold signal, located at the TGN; enlarged inset show [file pgen.1007795.s006.pdf]

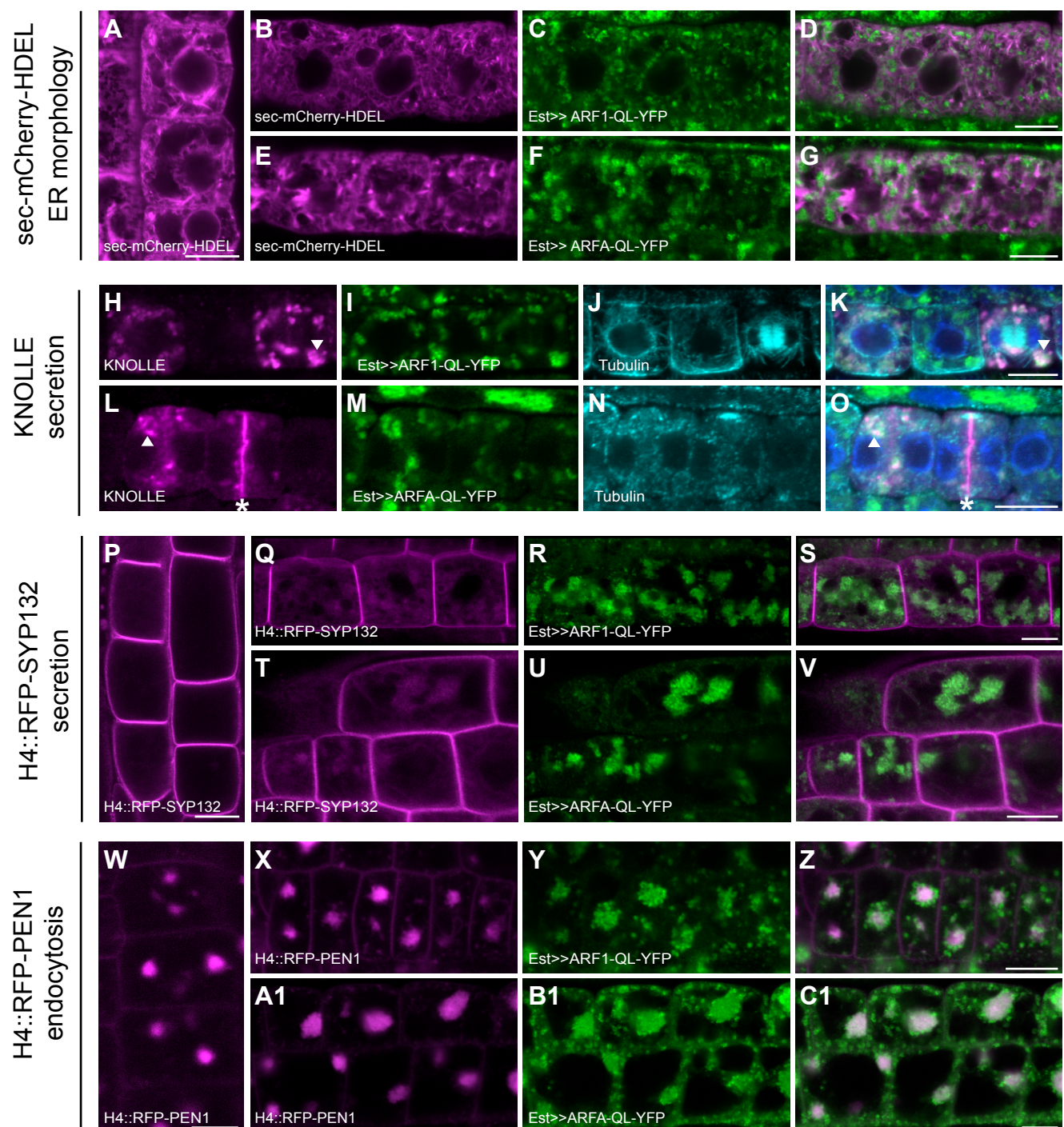

**Supplementary Figure S7**

Supplement: S7 Fig — Expression of hydrolysis-impaired, YFP-tagged (Q71L; QL; green) variants of ARF1 and ARFA was induced by 20μM estradiol for 5-6h and trafficking markers (magenta) were analyzed in live-cell imaging (A-G; P-C1) or immunostaining (H-O). (A-G) The ER was marked by sec-mCherry-HDEL (A, B, E). In ARF1-QL-YFP (B-D) and ARFA-QL-YFP (E-G) ER morphology was comparable to the wild-type control (Col; A). (H-O) The cytokinesis-specific syntaxin KNOLLE localized at the cell plate (asterisk) in some cells of ARFA-QL-YFP (L-O) expressing lines. Tubulin staining visualized phragmoplasts during cytokinesis (J, N; cyan). KNOLLE was not transported to the cell plate but localized in intracellular patches in some cells of ARF1-QL-YFP (H-K, arrowhead) and not in ARFA-QL-YFP lines (L-O; arrowhead). Blue, DAPI-stained nuclei. (P-V) SYP132 expressed from the Histone 4 promoter (H4::RFP-SYP132) localized at the plasma membrane in wild-type (Col; P), ARF1-QL-YFP (Q-S) and ARFA-QL-YFP (T-V). In addition, intracellular accumulation of RFP-SYP132 was observed in ARF1-TN-YFP (Q-S) and ARFA-QL-YFP (T-V) lines but not in wild-type (P). (W-C1) BFA treatment (1h 50μM) was used to visualize endocytosed RFP-PEN1 (Y, X, A1) in BFA compartments. Endocytosis of PEN1 was comparable to wild-type control (W) in ARF 1-QL-YFP (X-Z) and ARFA-QL-YFP (A1-C1). Scale bar, 10μm. The same wild-type controls were used as in Figs 3 and 5 and S3. (PDF) [file pgen.1007795.s007.pdf]

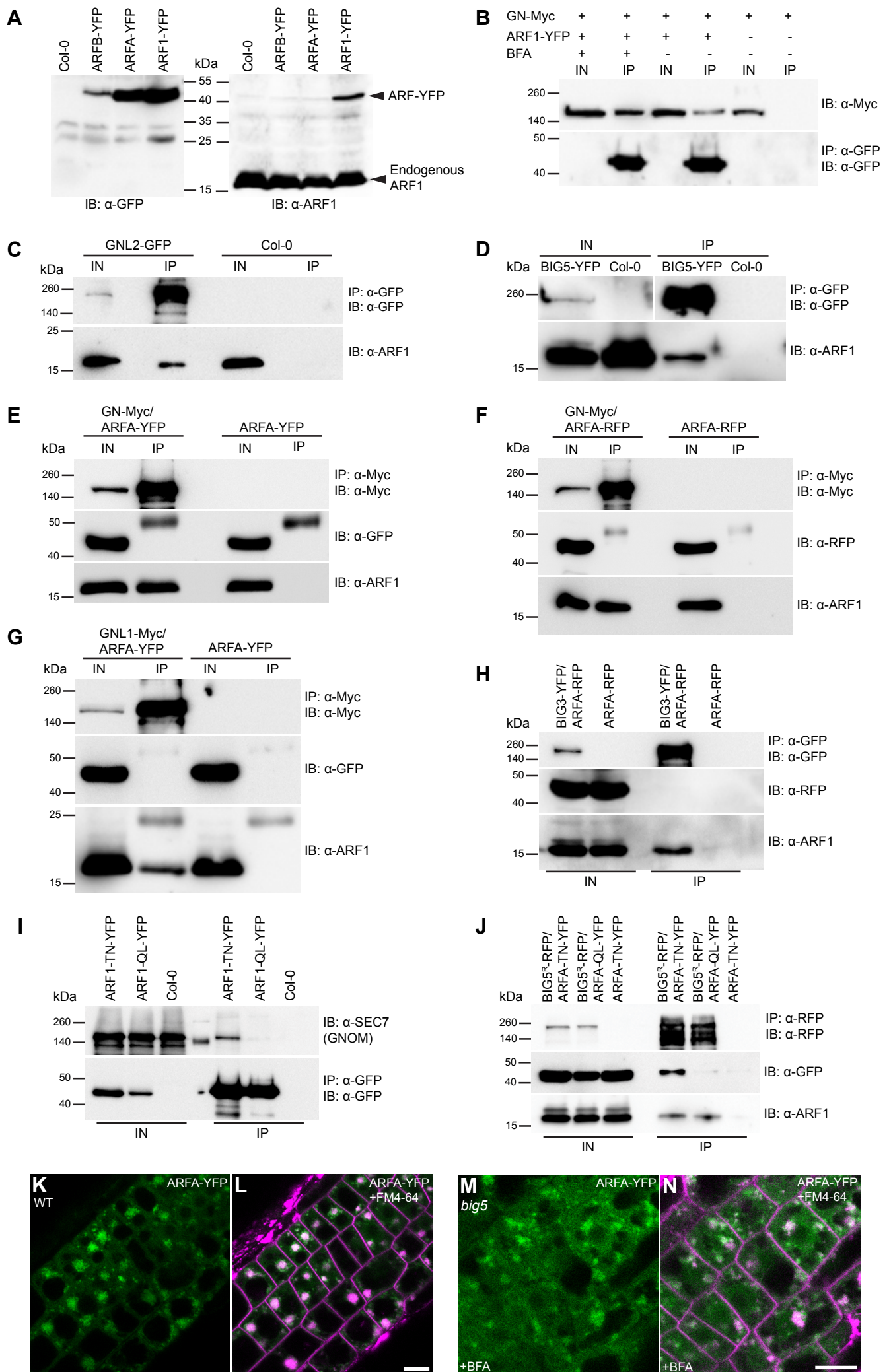

Supplementary Figure S8

Supplement: S8 Fig — (A) The α-ARF1 antiserum did not recognize ARFA and ARFB. Total protein from wild-type (Col-0) and the transgenic plants expressing ARF1-YFP, ARFA-YFP and ARFB-YFP were separated on SDS-PAGE followed by western blot using α-ARF1 (IB: α-ARF1) or α-GFP (IB:α-GFP) antibody. (B) GNOM-ARF1 interaction was enhanced in the presence of Brefeldin A (BFA). Immunoprecipitation (IP) was performed using GFP-Trap-agarose beads (IP: α-GFP) from transgenic plants co-expressing ARF1-YFP and GNOM-Myc (GN-Myc), in the presence or absence of BFA. IP was followed by immunoblot (IB) analysis using α-Myc antibody. (C) Co-immunoprecipitation of ARF1 with GNL2. Immunoprecipitation (IP) of GNL2 using GFP-Trap-agarose beads (IP: α-GFP) from transgenic plants expressing GNL2-GFP under the control of the GNOM promoter (GN::GNL2-GFP), was followed by immunoblot (IB) analysis using α-ARF1 antibody. Col-0, wild-type control. (D) Co-immunoprecipitation of ARF1 with BIG5. Immunoprecipitation (IP) of BIG5 using GFP-Trap-agarose beads (IP: α-GFP) from transgenic plants expressing BIG5-YFP was followed by immunoblot (IB) analysis using α-ARF1 antibody. Col-0, wild-type control. (E) ARFA (ARFB1b)-YFP did not co-immunoprecipitate with GNOM. Immunoprecipitation of GNOM-Myc (GN-Myc) from transgenic plants co-expressing ARFA-YFP and GNOM-Myc using α-Myc-agarose beads was followed by immunoblot (IB) analysis using α-GFP antibody and α-ARF1 antibody to detect YFP-tagged ARFA and endogenous ARF1, respectively. The protein band near 50 kDa in the IP lane corresponds to Ig heavy chain from α-Myc-agarose. (F) ARFA (ARFB1c)-RFP did not co-immunoprecipitate with GNOM. Immunoprecipitation(IP) of GNOM-Myc (GN-Myc) from transgenic plants co-expressing ARFA-RFP and GNOM-Myc was performed with α-Myc-agarose beads followed by immunoblot (IB) analysis using α-RFP antibody and α-ARF1 antibody. (G) ARFA (ARFB1b)-YFP did not co-immunoprecipitate with GNL1. Immunoprecipitation of GNL1-Myc from transgenic plants co-expressing [file pgen.1007795.s008.pdf]

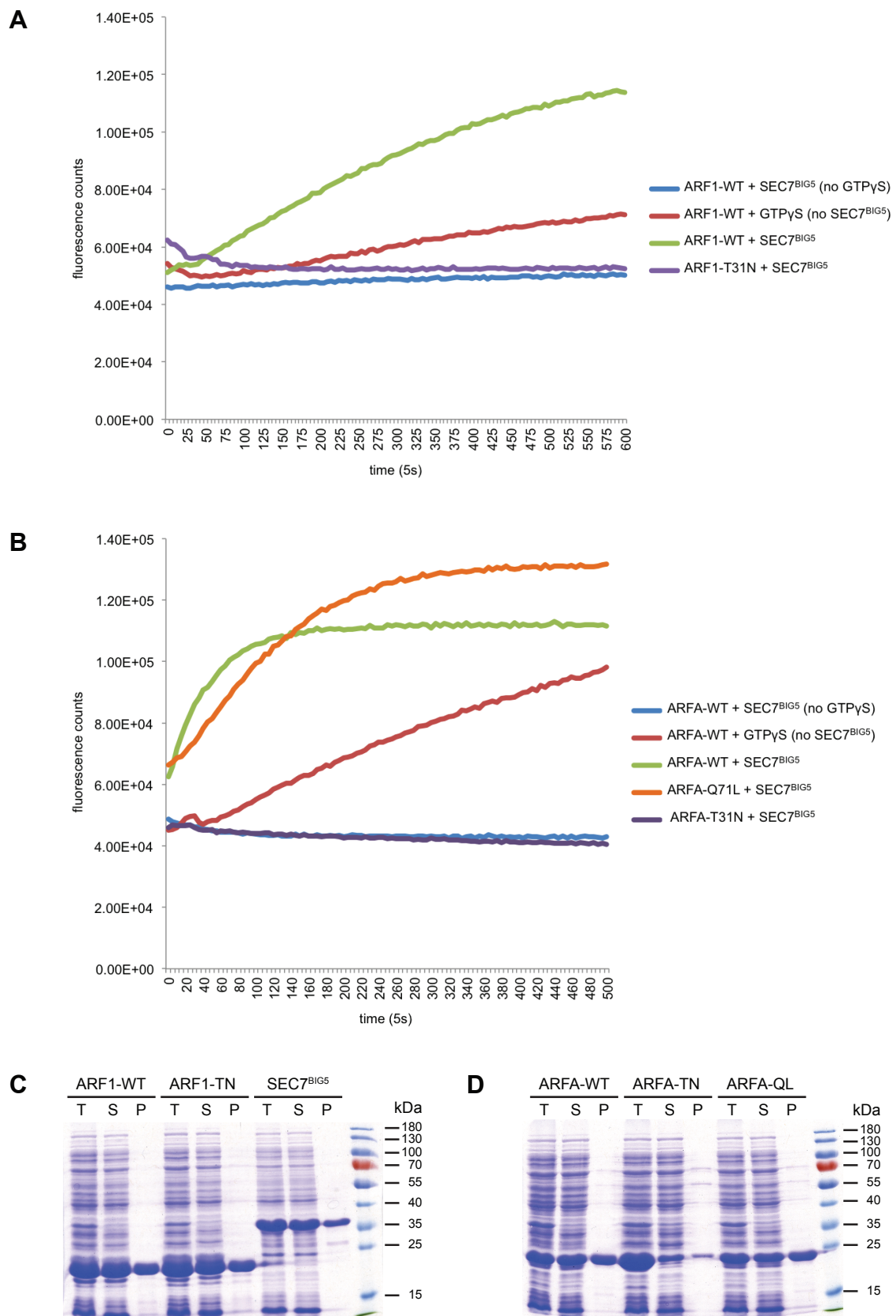

Supplementary Figure S9

Supplement: S9 Fig — (A-B) Effect of catalytic SEC7 domain of BIG5 (SEC7BIG5) on nucleotide exchange rate of ARF1 (ARFA1c) isoforms (A) and ARFA (ARFB1b) isoforms (B). The increase in tryptophan fluorescence over time reflects a conformational change from GDP-bound state to GTPγS-bound state. The blue line represents exchange activity of GEF (SEC7BIG5) on ARF1 wild-type (ARF1-WT) (A) and ARFA wild-type (ARFA-WT) (B) in absence of GTPγS whereas the red line represents spontaneous exchange activity of ARF1-WT (A) and ARFA-WT (B) in absence of GEF (SEC7BIG5). The green line shows GDP-GTP exchange activity of SEC7BIG5 on ARF1-WT (A) and ARFA-WT (B) whereas the purple line represents exchange activity of SEC7BIG5 on ARF1-T31N (A) and ARFA-T31N (B). The orange line shows GDP-GTP exchange activity of SEC7BIG5 on ARFA-Q71L protein (B). The experiments were performed using 1μM ARF, 50nM SEC7BIG5 and 66μM GTPγS at 37°C. The tryptophan fluorescence was measured at an interval of 5 seconds, using the excitation and emission wavelength of 298nm and 340nm, respectively. Note that both ARF1-T31N and ARFA-T31N do not show any increase in tryptophan fluorescence during the course of measurement. (C-D) Coomassie-stained SDS-PAGE showing expression and purification of His (6X)-tagged ARF1-WT, ARF1-T31N (TN), SEC7BIG5 (C) and ARFA-WT, ARFA-T31N (TN), ARFA-Q71L (QL) (D) using Ni-NTA resin. T, total; S, soluble fraction; P, purified protein (from Ni-NTA resin); kDa, kilodalton. See also S10 Data. (PDF) [file pgen.1007795.s009.pdf]
