# Supplementary material for: A single class of ARF GTPase activated by several pathway-specific ARF-GEFs regulates essential membrane traffic in Arabidopsis
Source: PLoS Genet. 2018 Nov 15;14(11):e1007795. doi: 10.1371/journal.pgen.1007795 (PMC6264874; doi:10.1371/journal.pgen.1007795)
Supplement: S1 Table — (DOCX) [file pgen.1007795.s010.docx]

**Table S1:** Evolutionary distribution of ARFs and ARLs within the Archaeplastida: isoform numbers and sequence identity to Arabidopsis proteins

| **ARF, ARL** | **Arabidopsis^a^**  (Brassicaceae) | **Eutrema**  (Brassicaceae) | **Carica**  (Brassicales) | **Theobroma**  (Malvales) | **Aquilegia**  (basal eudicots) | **Spirodela**  (monocots) | **Amborella**  (basal angiosperms) | **Marchantia**  (mosses) | **Klebsormidium**  (algae) |
| --- | --- | --- | --- | --- | --- | --- | --- | --- | --- |
| ARF1 | 6 isoforms  ARFA1a-f (At1g23490, At5g14670, **At2g47170***, At1g70490, At3g62290, At1g10630) | 8 isoforms  (>98% id.) | 4 isoforms  (2 99% id.,  1 90% id.,  1 83% id.) | 5 isoforms  (4 98% id.,  1 86% id.) | 6 isoforms  (4 97% id.,  1 87% id.,  1 63% id.) | 4 isoforms  (3 97% id.,  1 80% id.) | 5 isoforms  (4 97% id.,  1 86% id.) | 8 isoforms  (3 >94% id.,  2 67% id.,  2 59% id.,  1 51% id.) | 4 isoforms  (1 96% id.,  1 71% id.,  1 60% id.,  1 59% id.) |
| ARFA | 2 isoforms  ARFB1b (At5g17060)  ARFB1c (At3g03120 | 3 isoforms  (97% id.) | 1 isoform  (93% id.) | 1 isoform  (92% id.) | 1 isoform  (90% id.) | 1 isoform  (91% id.) | 1 isoform  (89% id.) | 2 isoforms  (82% id.,  50% id.) | 1 isoform  (74% id.) |
| ARFB | 1 isoform  ARFB1a (At2g15310) | 1 isoform  (92% id.) | 1 isoform  mixed identity  (ARFB 73% id.,  ARF1 70% id.) | 1 isoform  mixed identity  (ARFB 60% id.,  ARF1 56% id.) | Not present | Not present | Not present | Not present | low mixed identity  (ARL2 37%,  ARFB 40%) |
| ARFD | 2 isoforms  AtARFD1a (At1g02440)  AtARFD1b (At1g02430 | 1 isoform  (78% id.) | Not present | Not present | Not present | Not present. | Not present | Not present | Not present. |
| ARL1 | 1 isoform  ARF3/ ARFC1b  (At2g24765) | 4 isoforms  (96% id.) | 1 isoform  (93% id.) | 1 isoform  (95% id.) | 1 isoform  (92% id.) | 1 isoform  (90% id.) | 1 isoform  (90% id.) | 1 isoform  (88% id.) | 2 isoforms  (85% id.,  40% id.) |
| ARL2 | 1 isoform  ARLC1/HAL  (At2g18390) | 1 isoform  (98% id.) | 1 isoform  (81% id.) | 1 isoform  (93% id.) | 1 isoform  (90% id.) | 1 isoform  (87% id.) | 1 isoform  (86% id.) | 1 isoform  (77% id.) | 2 isoforms  (75% id., 55% id.) |
| ARL5 | 1 isoform  ARFC1/ARFC1a (At3g22950) | 1 isoform  (95% id.) | 1 isoform  (91% id.) | 1 isoform  (91% id.) | 1 isoform  (90% id.) | 1 isoform  (89% id.) | 1 isoform  (92% id.) | 1 isoform  (73% id.) | 1 isoform  (85% id.) |
| ARL8 | 4 isoforms  ARLA1a-d (At5g37680, At3g49860, At3g49870,  At5g67560) | 3 isoforms  (100% id., 97% id., 93% id.) | 2 isoforms  (94% id., 93% id.) | 1 isoform  (94% id.) | 3 isoforms  (95% id., 90% id., 88% id.) | 2 isoforms  (86% id.,  88% id.) | 2 isoforms  (87% id.) | 1 isoform  (81% id.) | 1 isoform  (81% id.) |
| ARFRP1 | 1 isoform  ARLB1 (At5g52210) | 1 isoform  (93% id.) | 1 isoform  (72% id.) | 1 isoform  (83% id.) | 1 isoform  (79% id.) | 1 isoform  (81% id.) | 1 isoform  (77% id.) | 1 isoform  (68% id.) | 1 isoform  (60% id.) |

^a^ Protein designations after Vernoud et al. (2003); Arabidopsis gene identifiers in brackets; id., identity of amino acid sequences (AtARFs or AtARLs, 100%).

* At2g47170 (ARFA1c; designated ARF1A1C in the TAIR database), used for raising ARF1-specific antiserum.
